# Application of nano‐radiosensitizers in combination cancer therapy

**DOI:** 10.1002/btm2.10498

**Published:** 2023-02-10

**Authors:** Mohammad Varzandeh, Leila Sabouri, Vahid Mansouri, Maliheh Gharibshahian, Nima Beheshtizadeh, Michael R. Hamblin, Nima Rezaei

**Affiliations:** ^1^ Department of Materials Engineering Isfahan University of Technology Isfahan Iran; ^2^ AmitisGen TECH Dev Group Tehran Iran; ^3^ Regenerative Medicine Group (REMED) Universal Scientific Education and Research Network (USERN) Tehran Iran; ^4^ Gene Therapy Research Center, Digestive Diseases Research Institute, Shariati Hospital, Tehran University of Medical Sciences Tehran Iran; ^5^ Student Research Committee School of Medicine, Shahroud University of Medical Sciences Shahroud Iran; ^6^ Department of Tissue Engineering School of Advanced Technologies in Medicine, Tehran University of Medical Sciences Tehran Iran; ^7^ Laser Research Center, Faculty of Health Science University of Johannesburg Doornfontein South Africa; ^8^ Network of Immunity in Infection, Malignancy and Autoimmunity (NIIMA) Universal Scientific Education and Research Network (USERN) Tehran Iran; ^9^ Research Center for Immunodeficiencies Children's Medical Center, Tehran University of Medical Sciences Tehran Iran; ^10^ Department of Immunology School of Medicine, Tehran University of Medical Sciences Tehran Iran

**Keywords:** cancer treatment, combination therapy, high‐z nanoparticles, oxygen‐mimetics, radiosensitizers

## Abstract

Radiosensitizers are compounds or nanostructures, which can improve the efficiency of ionizing radiation to kill cells. Radiosensitization increases the susceptibility of cancer cells to radiation‐induced killing, while simultaneously reducing the potentially damaging effect on the cellular structure and function of the surrounding healthy tissues. Therefore, radiosensitizers are therapeutic agents used to boost the effectiveness of radiation treatment. The complexity and heterogeneity of cancer, and the multifactorial nature of its pathophysiology has led to many approaches to treatment. The effectiveness of each approach has been proven to some extent, but no definitive treatment to eradicate cancer has been discovered. The current review discusses a broad range of nano‐radiosensitizers, summarizing possible combinations of radiosensitizing NPs with several other types of cancer therapy options, focusing on the benefits and drawbacks, challenges, and future prospects.

**Abbreviations**
BrUdR: bromodeoxyuridine
BSO: buthiomine sulfoximine
CPP: cell‐penetrating peptide
CAD: computer‐aided design
DAMPs: danger‐associated molecular patterns
DEF: dose enhancement factor
DSB: double stranded break
EPR: enhanced permeability and retention
GSH: glutathione
Gy: gray
Hf: hafnium
HSPs: heat shock proteins
HMGB‐1: high mobility group box‐1 protein
IUdR: iododeoxyuridine
LSPR: localized surface plasmon resonance
MRI: magnetic resonance imaging
MOF: metal–organic framework
miRNAs: microRNAs
MGd: motexafin gadolinium
NPs: nanoparticles
NOS: nitric oxide synthase
NO: nitrogen oxide
OER: oxygen enhancement ratio
PFC: perfluorocarbon
PDT: photodynamic therapy
PS: photosensitizer
PTT: photothermal therapy
PEG: polyethylene glycol
RT: radiotherapy
RNS: reactive nitrogen species
ROS: reactive oxygen species
RB: Roussin's black
SSB: single stranded break
siRNAs: small interfering RNAs
SOD: superoxide dismutase
TdT: terminal deoxynucleotidyl transferase
TPZ: tirapazamine
TEM: transmission electron microscopy
TOF: turnover frequency
XMP: X‐ray‐mediated photosensitizers

## 1 INTRODUCTION

The last decade has seen many different kinds of nanoparticles (NPs) undergoing investigation for various types of cancer treatment, including drug delivery, gene therapy, photodynamic therapy, photothermal therapy, etc.^1^ Due to the size of the NPs (1–100 nm), they have a large surface area‐to‐volume ratio, which allows them to absorb substantial amounts of drugs and quickly disperse throughout the bloodstream. Their larger area endows them with unique features, and improves their mechanical, magnetic, optical, and catalytic properties and thus increases their broader medicinal use.^2^

The heterogeneity of cancer and the multifactorial nature of its pathophysiology has led to the investigation of many different treatment approaches. Although each of these approaches has shown some promising results both in the laboratory and in the clinic, no definitive treatment to eliminate cancer has yet been established.^3^ Therefore, researchers have attempted to combine two or more different methods into a single integrated approach to increase the success of treatment.^3^

Combination therapy is a treatment strategy that combines two or more therapeutic agents and is the cornerstone of today's cancer treatment.^4^ Combining various therapies to target cancer increases the effectiveness compared with each therapy used alone Therefore, it is crucial to find more effective methods for integrated therapy that are also economically viable.^4^, ^5^ Conventional cancer treatments non‐selectively target all actively proliferating cells, leading to the destruction of both healthy and cancerous cells and consequent toxicity.

For some types of cancer, the best treatment is a combination of surgery, radiation, and chemotherapy, and possible other drugs. Surgery or radiation therapy treats locally confined tumors, while chemotherapy drugs also kill cancer cells that have spread to distant sites. In some cases radiation therapy or chemotherapy is given before surgery (neoadjuvant) to shrink the tumor, thus improving the likelihood of complete surgical removal of the tumor.^4^ Radiation or chemotherapy after surgery (adjunctive therapy) is designed to destroy any remaining cancer cells.^6^ The stage and type of cancer govern the choice of the optimum type of treatment.^4^

Radiation therapy combined with either (or both) surgery or chemotherapy is the mainstay of cancer treatment. This involves the transfer of high intensity and accurate beams of ionizing radiation to tumor tissue, resulting in the death of tumor cells. The heterogeneous structure of a bulky tumor requires a high radiation dosage, which causes damage to healthy tissues.^7^

The term radiosensitizer refers to any agent that can increase the efficiency and effectiveness of radiotherapy. During this process, the existing limitations of radiotherapy are identified and targeted to address them. The present review covers a wide range of radiosensitizing agents and discusses the state‐of‐the‐art and future prospects. Many combination therapy strategies are discussed, including the advantages and disadvantages, challenges and future perspectives.

## 2 RADIOSENSITIZING AGENTS

The mechanism of radiotherapy is classified into two types: direct and indirect effects. Direct damage is caused by the interaction of ionizing radiation without any intermediaries for disrupting biomolecule structure. This mainly affects single‐stranded and double‐stranded DNA molecules. The indirect effects are caused by the ionization of water in human tissue to produce hydroxyl radicals and other reactive oxygen species (ROS) that can also damage biomolecules and DNA.^8^

Radiosensitization is a process that increases the sensitivity of cancer cells to damage caused by radiation exposure. At the same time, it reduces the potentially harmful effects on the molecular and cellular structures of the surrounding healthy tissue.^9^ Hence radiosensitizers are exogenous agents that increase the effects of radiation therapy. Over the past few years, there has been a significant interest in using advanced formulations to enhance the effects of radiotherapy, especially the use of metal‐based nanoparticles.^7^, ^8^, ^10^ Radiosensitizer agents can be subdivided into three categories based on their structure: small molecules; macromolecules; nanomaterials. In addition to small molecule and nano‐radiosensitizers, macromolecules such as miRNAs, proteins, peptides, and oligonucleotides are also able to increase radiation sensitivity.^9^

### 2.1 Small molecule radiosensitizers

**FIGURE 1:**
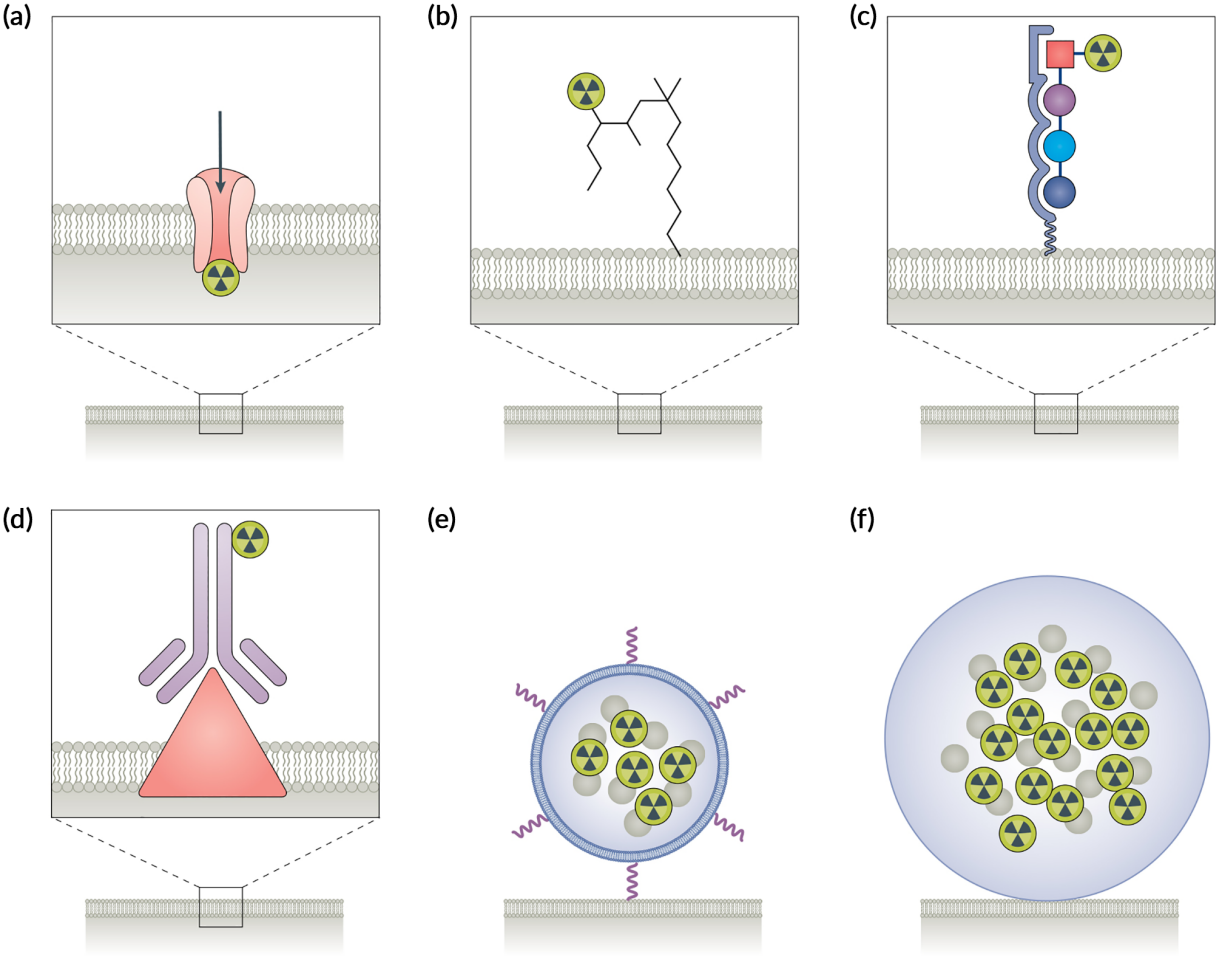
Constructs for radiopharmaceutical therapy. (a) radioactive elements; (b) small molecules; (c) peptide; (d) antibody; (e) nanostructure; and (f) microsphere, reprinted with permission from Reference 5.

Small molecules were studied at the very beginning of radiosensitizer discovery research.^11^ Subsequently, some small molecules were discovered that had promising results from the beginning and are now being used clinically (Figure 1).^5^, ^12^ Since then, a deep understanding of the molecular mechanisms of radiation therapy, and the signaling pathways associated with radiation sensitivity, have led to the production of drugs that act as radiosensitizers. Some of these can act on other pathways such as hypoxia‐response and cytokines.^12^, ^13^ Other types of chemical radiosensitizers, such as pseudo‐substrate, molecules that affect cell signaling, targeted transduction systems, and molecules that suppress radioprotective and repair properties, have also been developed, and some are in clinical trials.^13^ Cells in the process of division and undergoing DNA synthesis are unable to differentiate between thymine and its halogenated analogues, hence the newly synthesized DNA can act as a “pseudo‐substrate” ultimately leading to cell death.

Numerous signaling pathways related to apoptosis, metastasis, DNA repair, protein degradation, and other processes, can affect the effectiveness of radiotherapy. Small molecules that regulate vital pathways, such as DNA repair inhibitors and cell apoptosis activators, can enhance the efficiency of radiotherapy. Some compounds, such as the bioreductive drug Rsu1069, have dual or multiple effects, and can therefore sensitize cells not only to oxygen, but also disrupt signaling pathways and prevent DNA repair. With the advances in understanding of the mechanisms of radiation resistance, it became clear that multiple signaling pathways are associated with radiation sensitivity, providing more targets to improve the effectiveness of radiotherapy.^12^

Studies on radiosensitivity‐related pathways can provide new targets for radiosensitization protocols.^14^ Small molecule drugs are easily modified and have well‐understood evaluation systems for preclinical and clinical trials, which helps their rapid evaluation. Pharmacokinetics and pharmacodynamics are used in the design and screening of small‐molecule radiosensitizers to improve drug activity. The introduction of new methods such as computer‐aided design (CAD) and virtual screening has accelerated the development of radiosensitizers. In addition, emerging nanostructures and macromolecules, which act as radiosensitizers, have shown some promising results.^12^, ^14^

#### 2.1.1 Oxygen and oxygen‐mimetics

It is known that tumor cells located in a hypoxic microenvironment are more resistant to radiation than those in a normal oxygen microenvironment. The occurrence of hypoxia in the tumor microenvironment is one of the significant limitations of radiotherapy.^15^ The oxygen enhancement ratio (OER) or oxygen enhancement effect refers to increasing the therapeutic or destructive effect of ionizing radiation due to the presence of oxygen. This is called the oxygen effect, especially noticeable when cells are exposed to a dose of ionizing radiation.^12^, ^16^ Oxygen is a powerful radiosensitizer that enhances the formation of ROS and free radicals due to its electronic structure. After irradiation of an oxygenated tumor, energy transfer leads to radiolysis of water, with the initial formation of a radical ion, which after reacting with another water molecule, forms highly reactive hydroxyl radicals. Oxygen reacts with hydroxyl radicals to form peroxides, and then peroxides can cause permanent damage to cells and DNA.^9^, ^12^

**FIGURE 2:**
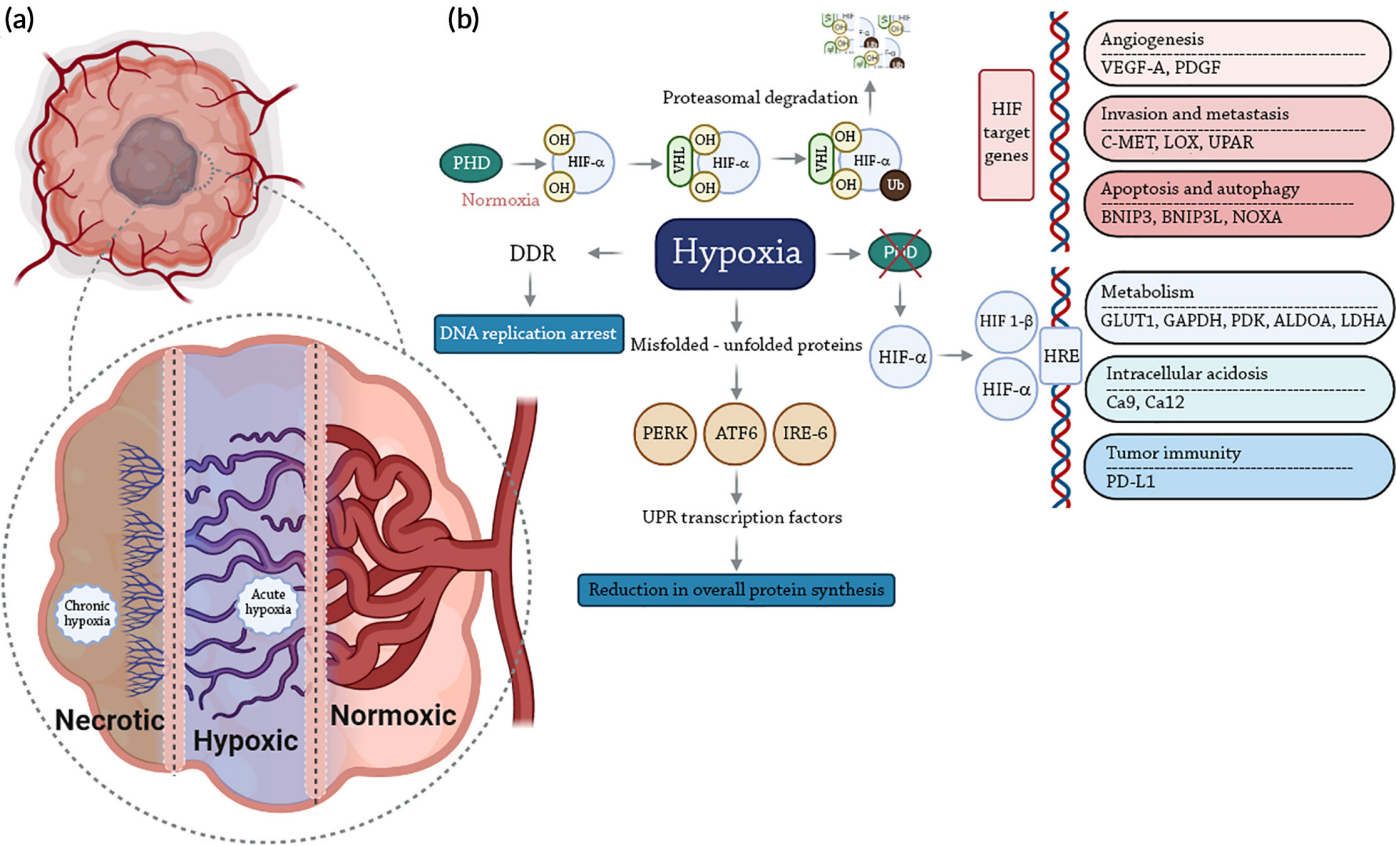
Tumor microenvironment and cellular pathways affected by hypoxia. (a) Oxygen concentration as a function of depth in tumor and (b) Sub‐cellular effect of oxygen concentration on cancer cells treated with radiation therapy.

The hypoxic conditions in the tumor microenvironment increase the resistance of cancer cells to damage by ROS and free radicals, and by altering signaling pathways to increase radiation resistance (Figure 2). For example, cells undergo apoptosis via the p53 pathway under normal conditions, while under hypoxic conditions, other interconnected pathways including HIF‐1α, VEGF, glucose transport, and glycolysis are activated.^7^, ^12^

Oxygen mimetics can imitate the chemical properties of molecular oxygen, yet they are designed to have higher electron affinity and better diffusion than oxygen. These oxygen mimetics can be introduced into the tumor environment, causing DNA damage and increasing cancer cell death during radiation therapy. Oxygen mimetics, also called true radiosensitizers, are of various types, and the most common types are nitrogen‐containing compounds, such as nitric oxide, nitrobenzene, nitroimidazole, etc.^7^

#### 2.1.2 Hypoxia‐specific compounds

Although oxygen and oxygen‐mimetics have been investigated for more than a decade, and significant progress has been made in these fields, they still face some obstacles and challenges.^17^ These compounds often produce free radicals under the influence of radiosensitizing incorporating oxygen atoms either in oxygen molecules or other nitro groups. Lack of tumor specificity, lack of different structure types, and side effects of using hyperbaric oxygen or active nitro groups necessitate further improvement and finding other kinds of radiosensitizers.

Due to the hypoxic conditions existing inside the tumor, agents that exert preferential toxicity under hypoxic conditions could be used as radiosensitizers. Some aromatic and aliphatic N‐oxides, quinones, transition metal complexes, and nitro‐compounds, have bio‐reductive properties, showed promising synergistic effects when combined with radiotherapy (RT).^18^ The most prominent agent in this group is tirapazamine (TPZ), which when reduced to its more active metabolites, induces double strand breaks (DSB), single strand breaks (SSB), and damage to nucleic acid bases in DNA of tumor cells under hypoxic conditions. Similarly, SN30000 a biosimilar analog of TPZ, can exert cytotoxicity after being metabolized by hypoxia‐activated reductases.^18^, ^19^, ^20^, ^21^

AQ4 (anthracenedione) is another agent with a high affinity for DNA which has shown promising results in preclinical and clinical trials.^22^ Its pro‐drug AQ4N, undergoes hypoxia‐sensitive reduction allowing it to act as a radiosensitizer in the hypoxic tumor environment.^23^, ^24^, ^25^ A class of nitro‐compound which are used as radiosensitizers is nitroimidazoles, particularly RSU1069 and its prodrug RB6145, with high electron affinity and ability to be reduced.^26^, ^27^ RSU1069 is a well‐known radiosensitizer which acts through electron reduction in hypoxic cells.^28^ RB6145 was developed after RSU1069 was found to show gastrointestinal toxicity, and has similar therapeutic effects but is more tolerable.^29^ The radiosensitizing effect of both drugs are improved when combined with photodynamic therapy or hyperthermia.^29^

#### 2.1.3 Pseudo substrates

The incorporation of compounds similar to nucleic acid bases into new DNA strands, leads to disruption in several vital processes of cells, especially DNA replication, and has been called pseudo substrates. The most prominent of these are halogenated analogs of nucleotides, such as bromodeoxyuridine (BrUdR) or iododeoxyuridine (IUdR). The proliferating cells in the tumor cannot distinguish between these compounds and natural thymidine, therefore they are incorporated into newly synthesized DNA molecules. Studies have shown a correlation between incorporation of BrUdR, number of DNA strand breaks, and clonogenic survival in the context of RT.^30^ 5‐FU (5‐fluorouracil), its prodrug capecitabine, and gemcitabine (20,20‐difluoro‐20‐deoxycytidine) are fluorinated analogs of nucleic acid bases, which are currently included in chemotherapeutic regimens. They exert their radiosensitizing effect through DNA damage and impaired DNA repair systems.^31^, ^32^

#### 2.1.4 Compounds influencing cell signaling pathways

Understanding the pathways responsible for the effects of radiosensitizing and radioprotective agents, can pave the way for the development of new tumor radiosensitizers, or radioprotective agents for normal tissue. Some of the pathways which have been identified in radiosensitization of tumors so far, include HDAC4,^33^ MDM2,^34^ c‐MET– PI3K–Akt,^35^ PI3K–Akt–mTOR,^36^ CSF1R,^37^ Wnt,^38^, ^39^ ADAM17,^40^ MAPK,^41^ RAD51,^42^ integrin α3,^43^ and integrin α6/Akt/Erk.^44^ Several drugs have been developed based on these pathways, such as TAS‐116, which inhibits heat shock protein (HSP) 90,^45^ HDAC inhibitors, which promote RT‐induced cell death and disrupt DNA strand repair,^33^ AMG 232, which inhibits MDM2 and suppresses tumor growth,^34^ and BKM120 and BEZ235, which target PI3K pathways and increase the sensitivity of tumor tissue to irradiation.^46^, ^47^ However, some of these drugs have more than one effect through different pathways, such as RSU1069, which inhibits DNA repair, along with the aforementioned hypoxia‐activated effects.

#### 2.1.5 Compounds suppressing radioprotective pathways

Mammalian cells contain a variety of reducing agents, which act to repair the damage induced by naturally produced free radicals. Glutathione (GSH) contains thiol groups and acts as an electron‐donating molecule, neutralizing free radicals inside cells.^48^, ^49^ Depletion of thiol groups could be another strategy for sensitizing tumors to RT. Binding to intracellular thiol groups using MnTE‐2‐PyP, or inhibition of thiol production by L‐S‐buthionine sulfoximine lowersthe GSH content of cells and promotes the effects of RT.^50^, ^51^, ^52^ Inhibition of other oxidoreductases, including superoxide dismutase (SOD), glutathione reductase, and thioredoxin reductase, could also impede DNA repair ability, and promote RT efficacy.^53^, ^54^, ^55^

### 2.2 Macromolecules

**FIGURE 3:**
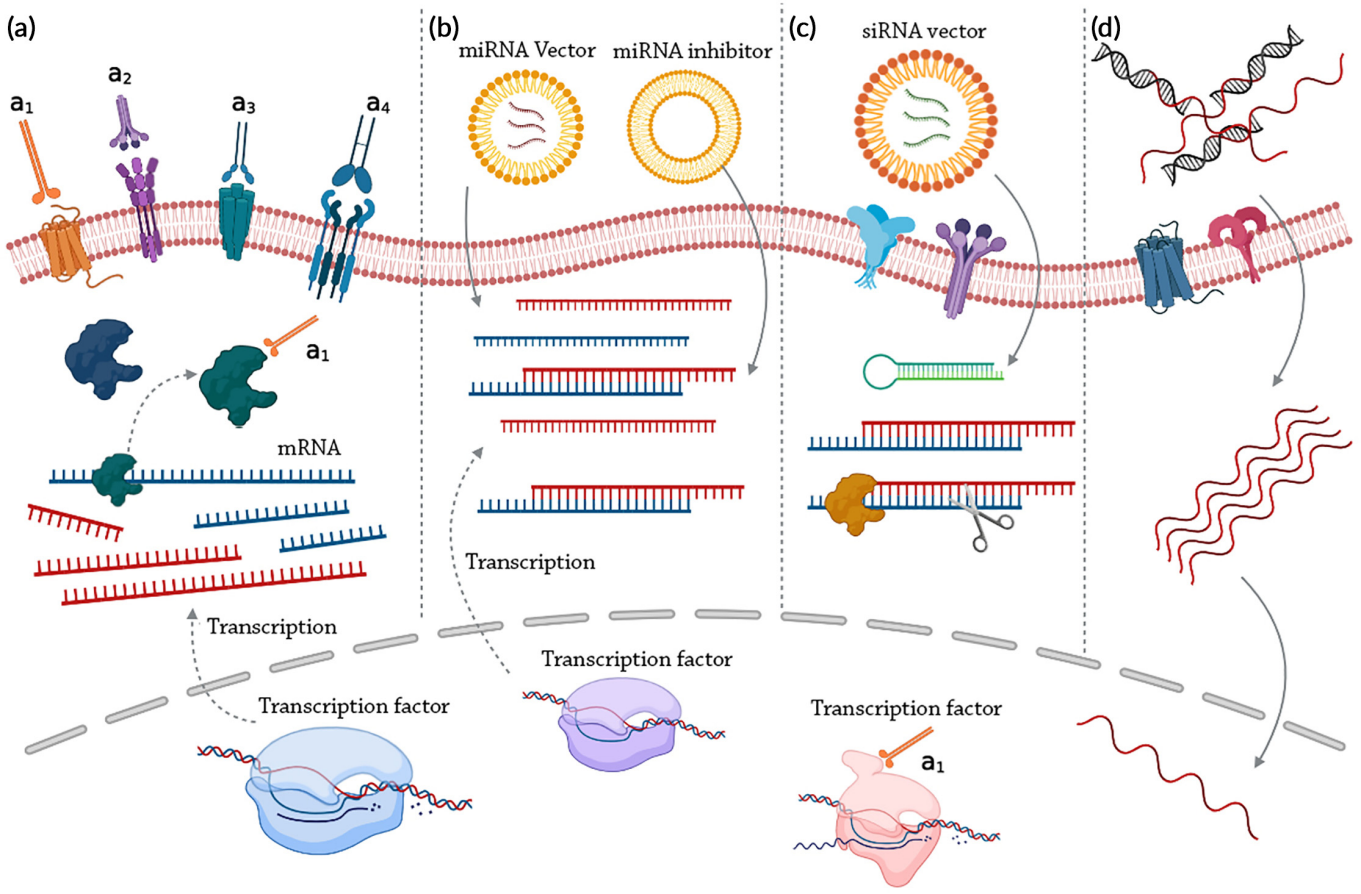
Macromolecules and radiosensitization. (a) Proteins and peptides: (a1) Direct binding between critical proteins; (a2) Radioactive seed loading; (a3) Delivery of radiosensitizers; (a4) conjugation with nanomaterials. (b) MiRNAs may attach to mRNAs to achieve radiosensitization: (Left) Downregulation through miRNA inhibitor; (right) Upregulation. (c) By binding to and destroying complementary mRNAs, siRNAs may increase radiosensitivity. (d) Oligonucleotides boost radiosensitivity by DNA binding.

Macromolecules including, microRNAs (miRNAs), small interfering RNAs (siRNAs), oligonucleotides, peptides, and proteins can all be used to alter the radiosensitivity of cells (Figure 3). Oligonucleotides can bind to DNA through complementary binding, while siRNAs and miRNAs can lead to gene silencing, and reduce the expression of DNA protective molecules or apoptosis inhibitors.^12^ Peptides and proteins can be used either for direct interaction with molecules responsible for radiosensitization, or for targeted delivery of radiosensitizer drugs.^12^

#### 2.2.1 MicroRNAs

miRNAs are small endogenous oligonucleotides (about 22 nucleotides long) which regulate gene expression at the post‐transcriptional level.^56^ Upregulation or downregulation of many miRNAs has been associated with radiosensitivity in multiple types of cancer (Table 1).^56^, ^57^, ^58^, ^59^, ^60^, ^61^ miR‐205 was found to be lower in radioresistant breast cancer, while upregulation of miR‐205 targeted ZEB1 and Ubc13 to improve radiosensitivity in preclinical models of breast cancer.^62^ Increased levels of miR‐34a contributed to more DNA damage via a p53 mediated pathway, while exogenous miR‐34a upregulation enhanced the radiosensitivity.^63^ Overexpression of miR‐1284 and miR‐203 produced higher radiosensitivity through regulation of Sp1 transcription and DNA repair pathways (e.g., JAK/STAT3 and Akt), respectively.^58^, ^64^, ^65^, ^66^ miR‐9,^67^ miR‐153,^68^ miR‐15,^69^ miR‐214,^70^ miR‐145,^71^ miR‐381,^72^ miR‐449a,^73^, ^74^ and miR‐200c^75^ are other microRNAs that have been reported to decrease radioresistance by affecting specific pathways in individual cancers.

**TABLE 1:** Effects of miRNAs, siRNAs, and oligonucleotides in cancer treatment and their mechanisms

Oligonucleotide miRs	Type of cancer	Mechanism	References
miR‐621	Hepatocellular carcinoma	Inhibiting SETDB1 and activating p53 signaling pathway	376
miR‐205	Breast cancer cells	Inhibiting DNA damage repair via targeting ZEB1 and the uniquitin‐conjugating enzyme Ubc13	62
miR‐144‐5p	Small‐cell lung cancer	Targeting activating transcription factor 2 (ATF2)	377
miR‐146a‐5p	Hepatocellular carcinoma	Activation of DNA repair pathway by replication protein A3	378
miR‐150	NK/T cell lymphoma	Inhibiting the AKT pathway	379
miR‐99a	Non‐small cell lung cancer	Inhibiting mTOR	380
miR‐139‐5p	Breast Cancer	Targeting multiple DNA Repair and ROS defense genes, including TOP2A, POLQ, RAD54L, TOP1, XRCC5, and MAT2A	381
miR‐320a	Colon cancer	Targeting p38 MAPK/JNK pathway and X‐linked inhibitor of apoptosis (XIAP)	382
miR‐1284	Hepatocellular carcinoma	Regulation of sp1 transcription	383
miR‐203	Glioma cells	DNA repair pathways (e.g., JAK/STAT3 and Akt)	65
miR‐9	Non‐small cell lung cancer	Inhibition of PI3K and phosphorylation of NF‐κB, P38 MAPK, Erk1/2 and Akt.	384
miR‐153	Glioma stem cells	Increased ROS production and decreased stemness through targeting Nrf‐2/GPx1/ROS pathway	68
miR‐15	Breast cancer	Targeting G2/M checkpoint proteins	69
miR‐214	Non‐small cell lung cancer	Regulation of p38MAPK	70
miR‐145	Cervical cancer	Targeting DNA damage repair‐associated genes, Helicase‐like transcription factor (HLTF)	71
miR‐381	Esophageal squamous cell carcinoma	Decreased tumor growth via targeting several genes	72
miR‐449a	Lung adenocarcinoma	Targeting multiple genes responsible for apoptosis and down‐regulating histone deacetylase HDAC1	73
miR‐449a	Prostate cancer	Downregulation of c‐Myc	74
miR‐200c	Malignant glioma, breast cancer, lung carcinoma	Downregulation of VEGF, HIF‐1α, and MMP2	75
miR‐126	Non‐small cell lung cancer	Decrease in PI3K‐Akt pathway activity	56
miR‐19b‐3p	Nasopharyngeal carcinoma	Activating the TNFAIP3/NF‐κB axis	61
miR‐34a	Glioblastoma multiform	Downregulating 53BP1, promoting p53‐mediated apoptosis	63
miR‐203	Gastric cancer	Downregulating ZEB1	385
miR‐374	Pancreatic cancer	Pathways other than X‐ray radiosensitivity	386
miR‐424	Cervical cancer	Targeting aprataxin, a DNA repair protein	387
miR‐18a‐5p	Lung cancer stem‐like cells	Downregulating both ATM and HIF‐1α	388
miR‐205	Prostate cancer	DNA damage repair impairment through inhibition of PKCε and ZEB1	389
miR‐138‐2‐3p	Laryngeal cancer stem cells	Up‐regulation of p38 expression and MAPK pathway activity	390
miR‐24	Nasopharyngeal carcinoma	Targeting Jab1/CSN5 function	391
miR‐101	Non–small cell lung cancer	Reduction in ATM and DNA‐PKcs levels	392
miR‐32‐5p	Colorectal cancer	Targeting TOB1 protein	393
miR‐203a‐3p	Ovarian cancer	Targeting ATM	394
miR‐421	Neuroblastoma	Suppressing ATM expression	395
miR‐155	Breast cancer	Repressing RAD51	396
miR‐1245	Breast cancer	Suppressing translation of BRCA2 and upregulation of c‐myc	397
*siRNAs*			
HIF‐1α siRNA	Prostate cancer	Redirecting aerobic glycolysis toward mitochondrial oxidative phosphorylation, leading to cell death through overproduction of ROS	77
HuR siRNA	Triple‐negative breast cancer	Increased ROS production and inhibition of thioredoxin reductase	80
S100A4 siRNA	Non‐small‐cell lung cancer	Upregulated p53 expression and E‐cadherin	398
NBS1‐siRNA	Non‐small cell lung cancer	Suppression of DNA repair and/ or X‐ray‐induced cell survival signaling pathways through NFKB and XIAP	399
survivin‐siRNA	Head and neck squamous cell carcinoma	Down‐regulation of survivin expression	79
*Antisense oligonucleotides*			
RNA subunit of telomerase	Melanoma, breast cancer, osteosarcoma	Inhibit telomerase	400
Human telomerase reverse transcriptase (hTERT)	Breast carcinoma	Promote radiation‐induced inhibition of telomerase	78
Cyclic AMP response element	Multiple cancer cell lines	Inhibition of cyclic AMP response element binding protein (CREB)	401
Human telomerase mRNA	Nasopharyngeal carcinoma	Inhibition of telomerase activity	402

#### 2.2.2 Oligonucleotides and siRNAs

Currently, siRNAs and antisense‐oligonucleotides, up to 25 nucleotides in length, can be rationally designed and efficiently synthesized. siRNAs are double‐stranded RNA molecules that can interfere with and degrade mRNAs after transcription.^76^, ^77^ siRNAs can be used for silencing the genes that contribute to radioresistance. An antisense oligonucleotide against telomerase reverse transcriptase is an example of the radiosensitizing function of oligonucleotides.^78^ Knockdown of survivin by siRNA negatively modulated the inhibition of caspase activation, leading to increased apoptosis after RT.^79^ Similarly, siRNA‐mediated knockdown of HuR mRNA resulted in enhanced radiosensitivity.^80^

#### 2.2.3 Protein and peptides

Many proteins and peptides have been used in cancer treatment, especially in the form of monoclonal antibodies or small peptides with affinity to tumor‐associated antigens. The anti‐cancer effects are produced by the inhibition of specific pathways, which can also affect the radiosensitivity of cells. For instance, SYM004 (epidermal growth factor receptor targeting antibody) could inhibit DSB repair and increase radiosensitivity through downregulation of the MAPK pathway.^41^ Similarly, the monoclonal antibody AIIB2 showed promising effects on head and neck squamous cell carcinoma by inhibiting DSB repair following inhibition of integrin β1.^81^

Another major application of proteins and peptides is the targeted delivery of drugs or radionuclides. Peptides have been used to precisely deliver radionuclides to tumors as a form of brachytherapy.^82^, ^83^ Radionuclides can be incorporated in a peptide receptor strategy.^84^, ^85^ Combining peptides for highly accurate drug delivery along with nanomaterials for a high drug loading capacity synergistically increases the effectiveness of the radiosensitizing agents.^86^ Peptides including DZ1, NKTR‐214, HSP‐70, and HMGB1 can all increase the radiosensitivity of tumors,^87^, ^88^, ^89^ while other peptides present in serum like C‐reactive peptide, HSP, and paraoxonase‐2 could be used as biomarkers for tumor radiosensitization.^90^, ^91^, ^92^, ^93^

### 2.3 nanomaterial radiosensitizers

**FIGURE 4:**
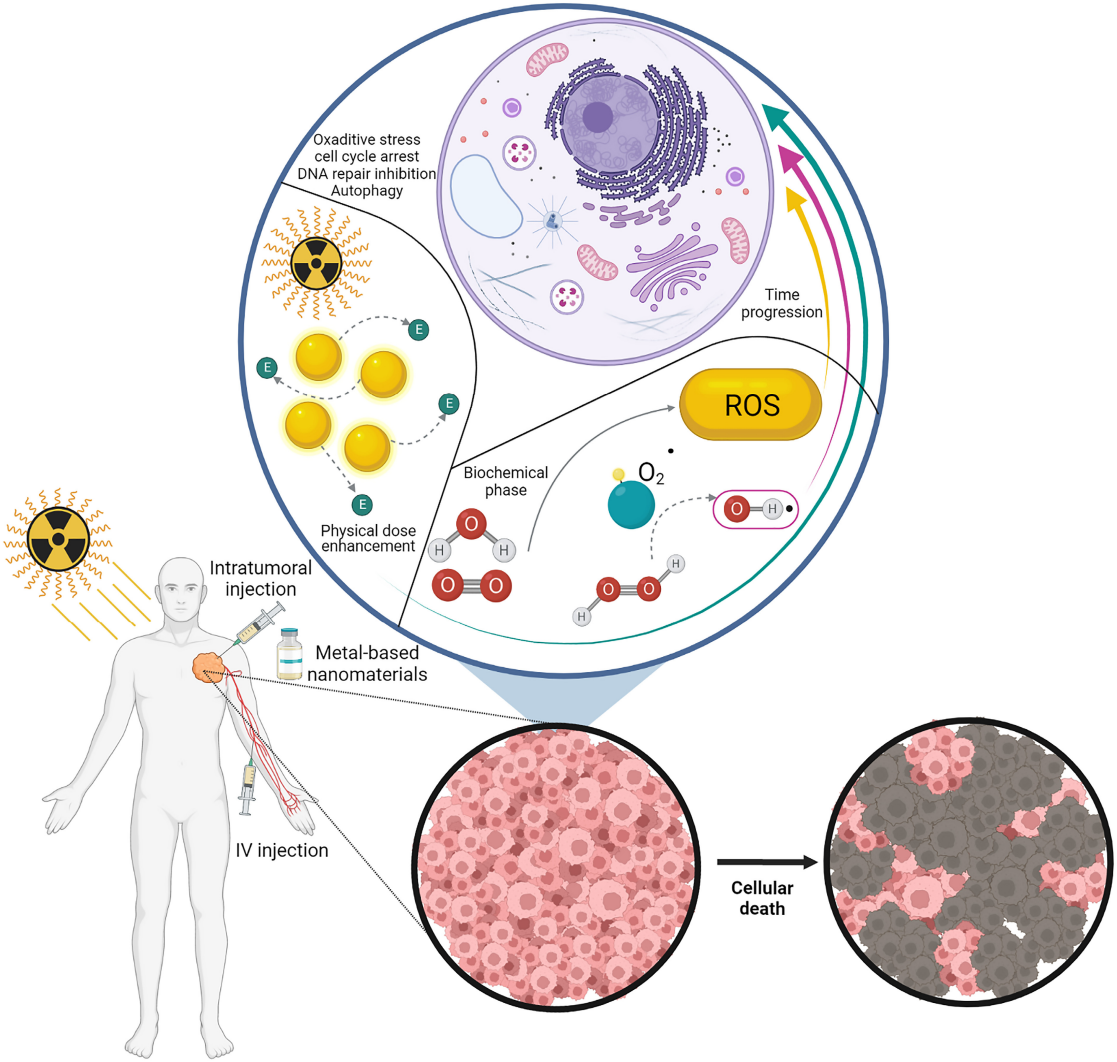
Schematically illustration of nano‐radiosensitizers utilization in vivo and its cellular effects

In recent years, utilizing various nanomaterial formulations (especially metal NPs) to enhance the tumor's radiation dose has increased significantly. Several nanomaterials such as metal NPs,^94^ quantum dots,^95^ superparamagnetic iron oxides,^96^ and non‐metallic NPs^97^ have been used to improve tumor radiation dose due to their unique physical and chemical properties. The use of nanomaterial radiation sensitizers is known as NP enhanced X‐ray therapy. These NPs are an excellent tool for cancer diagnosis,^98^ imaging,^99^ and treatment.^100^ Dense metal particles selectively scatter and absorb high‐energy rays such as gamma/X‐rays to better target the cellular components of tumor tissues. Although metal films and microparticles do not diffuse well in tumor tissue, NPs provide more cross‐sectional area to interact with radiation photons. Employing NPs reduces the dose of radiation therapy, and as a result, reduces damage to healthy tissue.^100^ Schematically representative of clinical trials on these nanomaterials following intravenous or intratumoral injection and subsequent cellular effect is shown (Figure 4).

Amongst multiple nanomaterials, integrating high‐z materials (such as gold, hafnium, bismuth, etc.) in cells causes a higher efficiency for cell damage caused by radiation. These NPs have chemical stability, slow metabolism, selective sensitivity, and significant effect at low doses.^101^, ^102^ For example, the cells grown on the gold film have increased the radiation dose several times. In addition, the injection of gold microparticles in tumor tissues caused a significant decrease in tumor growth after radiation.^7^, ^103^ Cell damage by high‐Z NPs is based on producing free radicals, secondary electrons, and reactive oxygen species (ROS).

The radiosensitizers based on drugs and macromolecules utilize the biological routes for sensitizing the cancer cells toward radiation. However, they are not able to profit from physical interaction of the high energy photon and any chemical reactions. High‐Z NPs could be designed and delivered to target cells or tumors to act as radiosensitizers, due to their high radiation absorption cross‐section compared with the surrounding soft tissue, increase the radiation dose received by the tumors.^7^, ^100^, ^104^ While the biological effects of the high‐Z nano‐radiosensitizers are limited (vs. physical enhancement), simultaneous administration of drug or macromolecular radiosensitizer will increase the treatment efficiency. Of note, utilizing pharmaceutical approaches for loading and active/passive release of the molecular and macromolecular radiosensitizers in nanoplatforms will enhance their efficiency. In section 3, high‐z nanoparticle will be discussed in detail.

## 3 HIGH‐Z NP‐BASED RADIOSENSITIZERS

As mentioned previously, radiotherapy is a common method of treating cancer that uses ionizing radiation to destroy tumor cells. However, some cancers are resistant to radiotherapy because of tumor heterogeneity and biological changes. Cell cycle alterations, hypoxia, cancer stem cells, inflammation, and DNA damage repair systems are factors that influence tumor resistance to radiotherapy.^105^, ^106^ The degree of radiation density in each tissue depends on the interaction of that tissue with the incident X‐rays, the density of electrons, and the amount of energy absorbed.^107^

High‐Z NPs can be designed to have photothermal, photoacoustic, and ionizing radiation absorption properties, while remaining chemically inert.^108^, ^109^ High‐Z NPs have some attractive properties, including low toxicity, easy preparation, easy surface functionalization, controllable size and morphology, and good chemical stability.^110^, ^111^ The effectiveness of these NPs was first observed in head and neck cancer patients who happened to also have metal implants, and who then underwent radiotherapy.^112^

Dose enhancement factor (DEF) is determined by dividing the deposited dose in the blank condition by the deposited dose amongst tumors harboring nano‐radiosensitizer. The three primary variables regulating the DEF were linked to the materials and pharmaceutical attributes (e.g., atomic number), incident radiation factors (e.g., photon energy), and subcellular localization of the nano‐radiosensitizer.^113^

Nano‐radiosensitizers featuring high‐Z elements promote cancer therapies via three principal mechanisms: physical, biological and chemical enhancement. In comparison with water, high‐Z materials contain significantly more electrons per atom, resulting in a greater attenuation cross‐section. In the other words, physical enhancement of the high‐Z elements refers to the absorbing more energy in comparison with water (in routine RT) endowing an enhanced electrons (Compton, photoelectric, and Auger) generation to the tumor site.

As discussed earlier, increased electron number production brings an enhanced direct and indirect effect on cancer cells which is studied under biological effects. In the other words, biological effects indicate the role of nano‐radiosensitizers on inducing oxidative stress, cell cycle modulations, bystander effects.^114^ In addition to the ROS production through physical enhancement, superficial atoms of the nanoparticles could act as a catalytic platform by transferring electron to the molecular oxygen.^115^ Due to the surface area increase followed by size reduction, gold nanoparticles with 3 nm size showed two‐fold turnover frequency (TOF) over 30 nm.^116^ Therefore, dense coating/functionalization on nano‐radiosensitizers restricts the chemical enhancement effect.

High‐Z metal NPs increase the local dose and focal ionization in surrounding cells through the photoelectric effect.^117^ The mechanism of how photons interact with high‐Z NPs is strongly related to the energy of the radiation beam, because the photoelectric effect decreases when the photon energy increases. With photons in the keV range, these NPs can increase the local radiation dose by 10 to 150 times relative to the surrounding soft tissue.^118^, ^119^ Therefore, keV energy photons should be used in combination with these NPs to optimize the radiosensitization effects.

**FIGURE 5:**
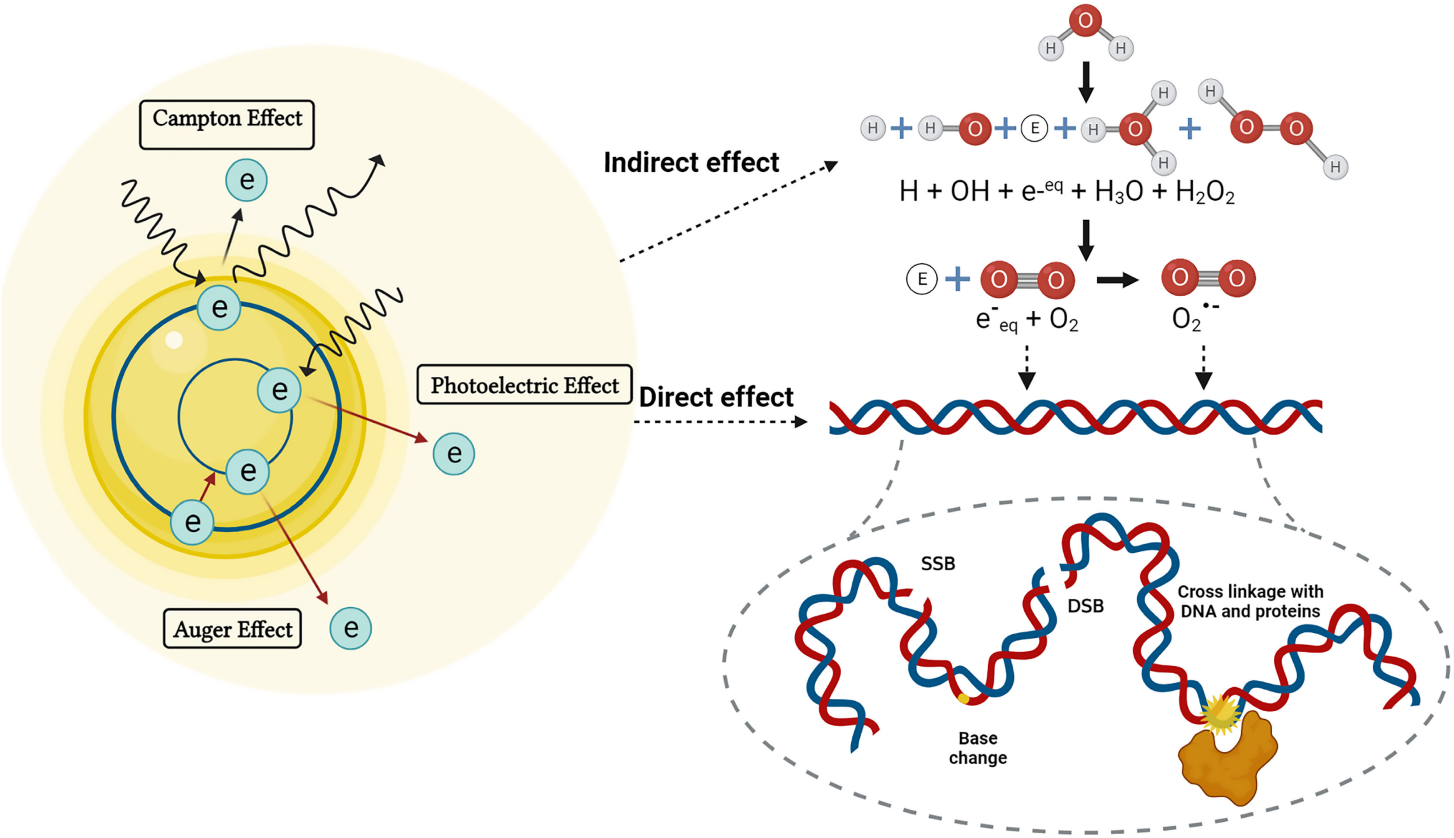
Auger, photoelectric, and Compton electrons mediated direct and indirect DNA aberration in nano‐radiosensitizers.

In photoelectric effect, the electron gets pulled out of the material if it receives a photon's energy and the photon has greater energy than the work function. Also, an atom emits a second electron when one of its internal electrons vanishes, a process known as an auger electron emission. In this effect, the second electron released is termed Auger electron. The process of the physical phenomena upon photon absorption, electron release and following DNA damage is represented in (Figure 5). The photoelectric effect, which predominates at low photon energy, may occur in subsequent to auger electron emission. In the other words, a cascade of low‐energy electrons that move over short distances and deposit their energy locally is generated by auger electron emission. These electrons can directly interact with biomolecules or produce ROS.^120^, ^121^, ^122^ Therefore, utilizing Auger electron for cancer treatment demands localization of nano‐radiosensitizers near to the nucleus.^123^ Using high‐Z elements with relatively low energy radiation (tens to hundreds of keV) is a promising way to treat resistant cancers. Loading the tumor with high‐Z elements produces a differentiating effect, by increasing radiation dose to the tumor and reducing it to the surrounding healthy unloaded tissue.^124^ On the other hand, photons in the keV range are generally not widely used in the clinic due to their low penetration depth. The low energy of Auger and photoelectric electrons is entrapped by other atoms of the nanoparticles.^125^

Conventional photons used in clinical radiotherapy have an energy in the range of 6–20 MeV.^126^ The probability of the photoelectric effect relative to the Compton effect and ion pair production decreases upon increasing the photon energy. Moreover, biological effects such as bystander, oxidative stress, and DNA damage can lead to radiosensitization with both MeV and KeV range photons. Interaction between radiation and high‐Z NPs produces significant levels of ROS, resulting in oxidative stress, DNA damage, and apoptosis.^114^, ^126^

In addition, while most high‐Z metal NPs cannot penetrate into the cell to affect the nucleus, but by increasing the physical dose, they can arrest the cell cycle, and DNA damage is caused by increased ROS production.^127^, ^128^ The targeting efficiency of high‐Z metal NPs is also important to determine the overall radiosensitizing efficiency. Today, various high‐Z NPs, such as hafnium oxide NPs, gadolinium oxide NPs, gold NPs, bismuth NPs, and silver NPs have all been studied for radiosensitization, each with their own unique feature. The next section will discuss the properties and function of these NPs.

### 3.1 Hafnium oxide NPs

Hafnium (Hf) is an element with high Z (Z = 72) and an electron emission ability that is used to produce X‐rays.^129^ The properties of this element include remarkable plasticity (stretchability), high‐temperature resistance, processability, and corrosion resistance.^130^ The radiosensitization ability of hafnium oxide NPs has been reported in several in vivo and in vitro studies.^8^, ^130^ Functionalized hafnium oxide NPs have a high electron density, which allows better absorption of incident ionizing radiation in order to deposit more energy in the tumor cells.^107^, ^131^ Exposure of Hf‐doped hydroxyapatite NPs to gamma rays increased the amount of ROS in the tissue and caused the death of cancer cells.^130^

Hafnium oxide NPs can accumulate in the cytoplasm of tumor cells, remain there for a long time, and deliver a high dose of energy into the cells.^107^ Therefore, hafnium oxide NPs have been investigated as a new approach to cancer treatment.^107^, ^132^ NBTXR3 (Nanobiotix, France) is a commercial product made of 50‐nm crystalline hafnium oxide NPs, functionalized by a negatively charged phosphate in aqueous solution (pH 6–8), which can be injected intratumorally and activated with external beam radiotherapy.^132^, ^133^, ^134^ In addition, hafnium oxide NPs are inert and have no toxicity to living cells, which made them promising in various clinical trials.^135^ The phase II/III trial showed positive results in patients with soft tissue sarcomas.^136^ Studies showed that radiotherapy‐activated NBTXR3 NPs also played an essential role in inducing an anti‐tumor immune response.^134^, ^137^ Radiotherapy plus NBTXR3 NPs can not only enhance the cell death caused by standard radiotherapy but can also activate further pathways for tumor cell death and immune response activation.

Maggiorella et al.^107^ injected NBTXR3 NPs into sarcoma bearing mice and then used cobalt 60 source radiation. They found that 24 h after NP injection and radiation, tumor growth was significantly inhibited compared with mice receiving radiotherapy alone. The crystal structure of these NPs did not change after a long time in vivo, which indicates the appropriate interaction of these NPs with ionizing radiation.

**FIGURE 6:**
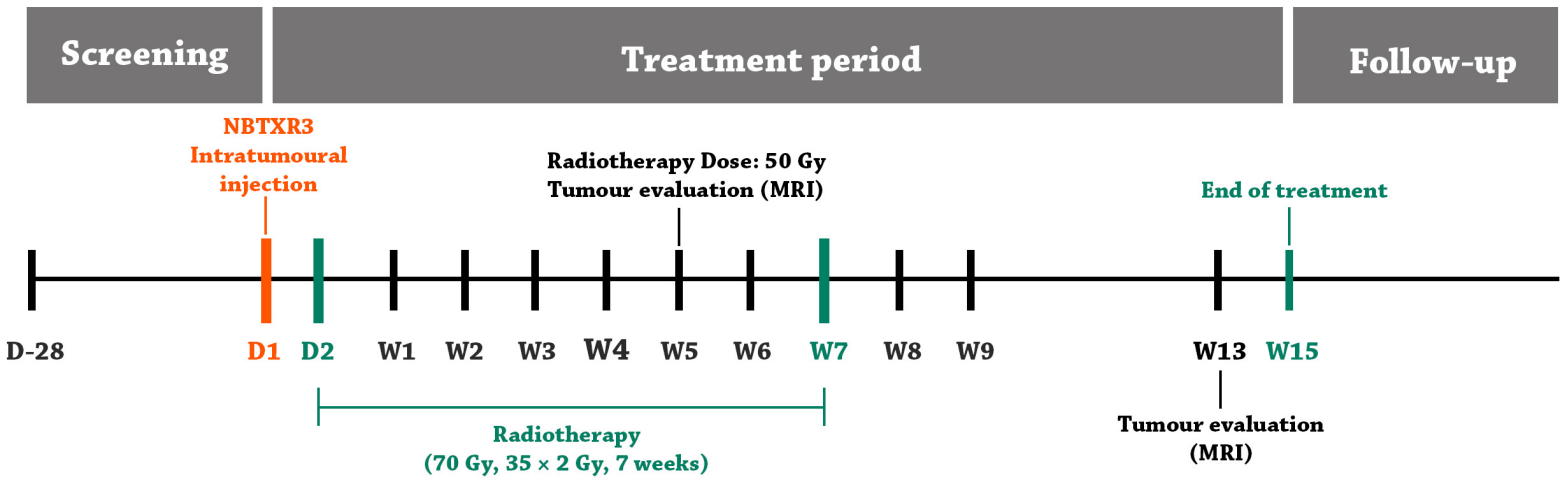
Diagram of radiation therapy treatment in clinical trial of NBTXR3 nanoparticles (W, week; D, day)

Hoffmann et al.^129^ used NBTXR3 to increase the energy deposition of radiation therapy to kill tumor cells in patients with locally advanced head and neck squamous cell carcinoma (LA‐HNSCC) (age 70 or 65) in a phase I clinical trial. NBTXR3 was injected intratumorally in patients, and then radiation therapy was performed. The dose of NBTXR3 was determined according to the tumor volume (Figure 6). They found that NBTXR3 remained stable and well‐dispersed in the tumor tissue during the treatment. Moreover, these NPs were not excreted in the urine, and did not leak into the tissue surrounding the tumor, and no side effects were observed. This study showed that NP amounts >10% of the tumor volume had a beneficial response over time, and was reported to be suitable for elderly or chemotherapy intolerant patients.^129^

### 3.2 Gadolinium oxide NPs

Gadolinium (*z* = 64) is another element with a high *z* atomic number with eight unpaired electrons, a + III oxidation state, and high coordination numbers between eight and ten.^138^ Gadolinium‐based NPs have been used in radiosensitization,^139^ neutron capture therapy,^140^ and as a contrast agent in magnetic resonance imaging (MRI).^141^ These nanoparticles are excreted in the urine within a few hours after intravenous injection (up to 30%).^142^ Several factors such as the clustering and heterogeneous distribution of gadolinium atoms, the widespread distribution of low‐energy electrons, and the optimal type of X‐rays can cause the radiation to be concentrated at the site and the tumor cells to be killed.^124^, ^143^ Using these NPs, a lower radiation dose concentration can be delivered to the nucleus than to the cytoplasm or membrane.^124^

Gadolinium‐based NPs have beneficial properties, such as high relativity, high biodistribution, passive uptake in tumors, and high permeability.^144^ Gadolinium interacts with various types of ionizing radiation, such as X‐rays, gamma rays, neutrons, or electrons, which could benefit its rapid translation to the clinic.^145^ Gadolinium‐based NPs display biocompatibility and a stable chemical composition, but the type of radiation used will affect their performance.^146^, ^147^, ^148^ The main limitation of utilizing these NPs is the similar ionic radius between gadolinium (III) and calcium (II), which leads to the potential replacement of calcium by gadolinium in bone.^149^, ^150^ In addition, the release of gadolinium could cause systemic nephrogenic fibrosis, therefore care must be taken in using these nanoparticles.^150^, ^151^

Motexafin Gadolinium (MGd) (Xcytrin, Sunnyvale, CA) is a compound of gadolinium that is used as a photosensitizer in photodynamic therapy for cancer, by producing ROS and inhibiting tumor growth. It also increases MRI signals, targets cancer cells (such as glioblastoma and brain metastases) and enhances cytotoxicity in combination with radiation. Motexafin gadolinium is currently being tested in clinical trials as a radiosensitizer.^152^, ^153^ Dotarem (Guerbet, France) is another commercial product based on gadolinium, which is used as a contrast agent in MRI.^154^ The safety and efficacy of Dotarem have been demonstrated in more than 7000 patients. Dotarem excretion occurs by glomerular filtration in the kidneys, or by peritoneal dialysis. In patients with renal insufficiency, Dotarem is slowly eliminated but still has good safety, however it can occasionally cause anaphylactic shock.^155^

Mignot et al.^156^ developed another type of gadolinium‐based nanoparticles called AGuIX (NH TherAguix, France). These NPs are about 5 nm in diameter and are rapidly excreted by the kidneys, allowing radiosensitization, and acting as contrast agents in MRI.^157^ The image‐guided therapy of the AGuIX alongside with its tumor accumulation via EPR effect, enabled a low healthy tissue damage.^158^, ^159^ Hu et al.^160^ used AGuIX radiation sensitivities to diagnose tumors and treat hepatocellular carcinoma (HCC) radiotherapy. Gadolinium in these NPs increased radiation dose deposition and radiation sensitivity. AGuIX was a favorable NP for conducting MRI and increasing radiation sensitivity; by increasing the tolerance of liver tumors to radiation, it promoted conventional radiotherapy methods in treating HCC. They showed that the presence of AGuIX significantly reduced HepG2 cell survival when combined with X‐rays.^160^ MRI imaging (in vivo) showed that the AGuIX NPs tumor/liver concentration ratio was highest 1 h after intravenous injection. The anti‐tumor effects of high‐dose AGuIX‐mediated radiotherapy were more effective. They concluded that AGuIX could increase the sensitivity to MRI and to radiotherapy in HCC.^160^

Wu et al.^161^ used hyaluronic acid‐functionalized gadolinium oxide NPs for MRI, and for radiosensitization of tumors. The resulting NPs were well dispersed in water, and showed low cytotoxicity, good biocompatibility, could be taken up by endocytosis into the cytoplasm of cancer cells mediated by hyaluronic acid receptors. The gadolinium oxide NPs allowed the radiosensitization of tumor cells by increasing apoptosis and arresting the cell cycle.^161^

### 3.3 Gold NPs

Gold nanoparticles have been extensively studied as tumor radiosensitizers due to their advantages including strong photoelectric absorption coefficient, good biocompatibility, and their high surface‐to‐volume ratio.^10^ Due to their enhanced permeability and retention (EPR) properties, gold nanoparticles can accumulate at the tumor site, but have low permeability to capillaries and normal blood vessels in other tissues, such as the heart. They can be used as an imaging contrast agent, in order to diagnose diseases and track treatments. Due to their controllable size, and unique chemical, electrical and optical properties, gold NPs have become a major candidate for use in biological applications.^10^, ^126^

In vivo research has shown that X‐ray RT combined with gold NPs acting as a radiosensitizer, can increase the survival of tumor bearing mice.^162^ Radiation plus gold NPs increased the formation of free radicals in cancer cells, and interrupted the cell cycle.^114^ The lethal effect of NPs as radiosensitizers depends on their size, while according to a previous report, gold NPs with a diameter of about 13 nm coupled with a radiation dose of 4 to 8 Gray (Gy) may have the best lethal effect.^7^, ^10^, ^126^ Gold NPs at a dose of 6 Gy provided the most lethal effects to inhibit tumor growth.^102^ Gold NPs larger than 30 nm showed the same effect as 13 nm, but at the same time, their toxicity was higher.^102^ Nanoparticles coated with polyethylene glycol (PEG) with a diameter of ~13 nm have been used to improve CT scan imaging and radiosensitivity with optimized results. Moreover, gold NPs can also be modified with several ligands to allow drug and gene therapy, and increase the biocompatibility of these nanoparticles.^102^

### 3.4 Bismuth NPs

Since bismuth has a high atomic number, low toxicity, and low cost, bismuth nanoparticles could act as diagnostic and therapeutic agents, while they have also attracted widespread attention as a design factor in radiation therapy and imaging.^163^ Bismuth‐based sensitizers have low toxicity, easy availability of NPs, and cost‐effectiveness as their advantages.^164^

As a biocompatible element with an atomic number of 83, bismuth can maximize the efficiency of radiation absorption, and has been clinically used for many years. Bismuth NPs have biodegradable properties and can be removed from the body as soluble ions.^165^, ^166^ Various synthetic methods have been developed to increase the efficiency of bismuth NP preparation, including thermal dissolution, photochemical, and precursor methods.^165^, ^166^, ^167^ Recently, folate‐modified bismuth NPs coated with erythrocyte membranes have been developed for breast cancer radiotherapy, especially to increase the generation of free radicals. In addition, cellulose nanofibers have been used to fabricate bismuth NPs, which increased the production and secretion of free radicals in the presence of X‐rays, resulting in good tumor destruction. Due to the presence of carbonyl groups on the bismuth nanofibers, they were effectively absorbed and local oxidation was prevented, making them biocompatible.^165^

### 3.5 Silver NPs

Silver NPs can kill cells by apoptosis, activation of oxidative stress, and induction of excessive membrane fluidity.^102^ Silver NPs have unique optical, electrical and antibacterial properties, and have been widely used in biosensors, photonics, electronics, and antimicrobial applications. The investigation of silver NPs in cancer treatment has yielded positive results.^168^, ^169^ The use of silver NPs as radiosensitizers, especially in the treatment of brain tumors, has been investigated with promising results. For example, silver NPs showed a better radiosensitizing effect versus gold NPs (at same molar mass) on glioma tumors, leading to increases in autophagy and apoptosis.^170^

However, hybrid combinations of silver NPs with other types of NPs can strengthen the sensitizing effect.^171^, ^172^, ^173^, ^174^, ^175^ In order to combine the different properties of separate NPs together, the easiest way is to coat them both in a suitable shell, such as silica.^176^ On the other hand, to design effective NPs for cancer radiation therapy, selective targeting of tumor cells using various strategies is important. One promising method to improve the accumulation of different NPs in tumor cells is the attachment of targeted ligands. Examples of ligands for this purpose include, antibodies, peptides, aptamers, and ligands for cancer cell surface receptors, such as folate or transferrin.^177^

## 4 QUANTIFYING RADIATION SENSITIZATION TECHNIQUES

The use of NPs changes the quality of radiation and creates complex patterns of ionization, which ultimately leads to fatal damage to cells.^177^ Multiple reports show the effect of NPs on cell cycle, metabolic activity and DNA repair pathways. These effects depend on a complex range of physical, chemical, and biological parameters of NPs such as size and type of material, charge, coating, reactive radical production and cell uptake rate, cell cycle, etc.^114^ On the other hand, the most widely used method in radiobiology to study the effectiveness of a treatment is the clonogenic method (or colony formation). A cell that has lost its ability to reproduce is considered dead. This type of assay is the most widely used method to evaluate the radiation sensitivity of various cell lines and is considered as the gold standard for determining the response to radiation.^178^, ^179^

Despite numerous researches related to the use of NPs with radiation, there is a few precise and appropriate guide to evaluate the effectiveness of NPs. Having a comprehensive list of procedures and clear guidelines on comparative quantification methods used to assess radiation increased due to the fact that NPs application would ameliorate the NP and radiobiological community to better understand NP‐mediated effects and translate NPs studies to the clinical stage.^180^, ^181^ The number of colonies formed after treatment is calculated as a function of radiation dose and provides survival curves for evaluation. In addition, alternative methods for estimating the survival fraction using viability experiments like methylthiazole tetrazolium (MTT) method and trypan blue exclusion test have been developed and used.^182^

Accordingly, a comprehensive approach to measure radiosensitivity based on DNA damage, oxidative stress, cell survival (such as apoptosis and autophagy), cell senescence and signaling is needed. Several other techniques for studying DNA damage, including immunocytochemistry and gel electrophoresis, which are significantly time‐saving, require uncomplicated skills and protocols, and are less expensive.^183^ However, transmission electron microscopy (TEM) is a unique facility for detecting the type and location of DNA damage. Approximately 10–50 cells are analyzed utilizing the TEM at a time; hence, this method provides a better quality than other techniques, while it could not determine the results accurately. TEM usually uses the immunogold‐labeling method to characterize DNA damage. Primary antibodies target specific repair proteins that are conjugated by gold secondary antibodies similar to immunocytochemistry.^184^ Although TEM has not been used to study DNA damage caused by radiation sensitivity, it was used to monitor cell uptake and distribution of NPs.^185^ Furthermore, recently the technical use of TEM has been thoroughly described as a tool to study NP‐induced radiosensitivity in vitro.^179^, ^185^

Furthermore, flow cytometry could be used to detect DNA damage and analyze the cell cycle.^186^ Propidium iodide is the most commonly used dye for quantitative assessment of DNA content and is a very useful technique for studying various checkpoints during the cell cycle.^187^ In this method, nucleotides were extracted from cells, then they were stained with ethidium bromide fluorescent dye, and then they were exposed to laser light in flow cytometry.^187^

Flow cytometry and TEM are also used to investigate cellular uptake and final localization of nanoparticles.^188^ Recently, electrophoresis‐based DNA fragmentation methods have been used to quantify DNA damage. DNA lesions can be detected by gel electrophoresis, a rapid method that determines the average density of breaks and types of DNA lesions in nanogram amounts.^180^ Electrophoresis, in turn, has different types.

Terminal deoxy nucleotidy dutp nick end labeling (TUNEL) staining, which is also called TUNEL assay, forms DNA breaks created when DNA is fragmented in the last stage of apoptosis.^189^ The enzyme terminal deoxynucleotidyl transferase (TdT) can label the rough ends of double‐stranded DNA breaks when binding deoxynucleotides to the hydroxyl ends of DNA breaks, while Nucleotides bound by TdT are stained with fluorescent dye.^189^ This method can be an alternative method for agarose gel electrophoresis to analyze the formation of DNA fragments during apoptosis.^179^, ^190^

Immunoblot or western blot is also used to detect changes in protein expression after treatment.^191^ In this method, protein expression is sampled at different time points after X‐ray irradiation to determine how NP pretreatment increases radiation sensitivity.^191^ This is done both in the presence and absence of NPs. Primary antibodies were considered based on apoptosis, DNA damage, repair and oxidative stress.^192^ The ROS is one of the possible causes of aging, while radiation could lead to an increased senescence phenotype. Since ROS production could be responsible for NP‐induced radiosensitization, it is necessary to investigate whether cells irradiated with NPs lead to increased senescence compared with cells irradiated alone.^193^

In general, the radiation sensitivity effects of NPs could be classified into three groups, including physical, chemical, and biological effects. Table 2 demonstrates the comparison amongst using radiotherapy with or without NPS. Biological effects refer to cell damage and include DNA damage or inhibition of DNA repair, cell cycle effects, and cell death. Radiosensitivity directly depends on the cellular and intracellular distribution of NPs, which may damage specific cellular components such as the cell membrane, cytoplasm, nucleus, mitochondria, and endoplasmic reticulum, as well as other organelles. Sometimes theoretical models differ from experimental studies, indicating that we still do not fully understand biologically driven processes.^114^ The radiosensitizing effect of NPs strongly depends on the absorption by cells and also on their intracellular distribution.

**TABLE 2:** The comparison between radiation therapy alone and combined with nanoparticles^180^

	Radiotherapy	Radiotherapy + NPs
IR dose	Higher doses of IR, leading to damage both in cancer and normal tissues	Lower doses of IR (0.5–3 Gy), leading to local NO‐induced radiosensitization of cancer tissue, minimizing damage to the surrounding normal tissue, simultaneously. Types of IR: kV photons, MV photons, particles
Oxidative stress/ROS	Producing radical species of OH, O_2_, H_2_O_2_, O^−2^ due to water radiolysis	Increased production of ROS and oxidative stress due to enhanced electron production near cellular organelles
DNA damage	SSBs, base lesions, AP sites, DSBs	Increased DNA damage (SSBs, DSBs) Increased possibility for complex DNA damage (clustered lesions) (difficulty in repair/misrepair/unrepaired)
DNA repair	SSBs, base lesions, AP sites (Easily repaired) DSBs (Difficulty in repair /misrepair)	NPs may prevent DNA repair protein synthesis or avoid recruitments to the nucleus Toxic metal ions produced from NPs may interfere with DNA repair (e.g., Ag NPs, CuO/ZnO NPs)
Toxicity	Only genotoxic effects due to radiation damage (e.g., ROS production)	Increased toxicity due to NP materials, high concentration (100–500 ug/ml), size, prolonged presence in the body (e.g., Ag NPs, Al NPs, TiO_2_ NPs, IO NPs, Cu NPs) or some of the above combinations
Outcome	Cell killing	Increased cell killing

Multiple factors affect the adsorption of a NP, such as material, size, shape, surface charge. The cellular uptake route for NPs is quite essential since it determines the fate of NPs, their lifetime and circulation inside the cell and their ability to reach specific targets or drug release. Usually, NPs enter the cell from the extracellular environment through endocytosis by vesicles that are produced from the cell membrane.^194^ Endocytosis is mainly divided into two categories: phagocytosis and macropinocytosis, and nanoparticles are mainly located in the cytoplasm inside vesicles and are often not located near the nucleus.^127^

## 5 SYNERGISTIC EFFECTS OF RADIOSENSITIZING NPS IN COMBINATION THERAPY

The effort to find more efficient methods to treat cancer has increased within the past few decades, leading to the emergence of novel modalities, such as photo‐thermal therapy, photodynamic therapy, immunotherapy, and of course radiation therapy. The tumor microenvironment contains a dynamic set of complex biological and molecular components with an established cross‐talk, leading to overall progression and eventually to metastasis. Cancer cells, vascular cells, cancer stem cells, and cancer‐associated fibroblasts and immune cells constitute the cellular populations of the tumor.^195^, ^196^ In contrast, the tumor ECM consists of collagen, elastin, laminin and fibronectin, which differs with cancer type and progression stage.^197^

Photothermal therapy (PTT) leverages the increased temperature caused by laser‐irradiation of light absorbing compounds or chromophores in the tumor to produce localized hyperthermia. The targeted administration of nanoparticles followed by laser irradiation provides locally controlled photo‐induced hyperthermia for precision tumor treatment.^198^ PTT can induce direct cell death, modify the tumor microenvironment, and alter intracellular pathways. Moreover, PTT can induce an immune response by releasing tumor antigens, which can be magnified even further in combination with immunotherapy such as checkpoint blockade inhibitors.^199^, ^200^, ^201^

Another technology based on laser radiation is photodynamic therapy (PDT) which produces cytotoxic reactive oxygen species (ROS) causing cancer cell death. PDT can employ organic photosensitizers (e.g., porphyrins, phthalocyanines, or dyes) or inorganic photosensitizer agents (e.g., semiconducting NPs or quantum dots). In both cases a photochemical reaction takes place upon laser irradiation leading to the generation of ROS.^202^

RT is a very common cancer therapy method all around the world. In this method, the high energy radiation is irradiated to the tumor to produce ROS following ionization, which interacts with cellular compartments leading to death.^203^ Recently, nanoparticle‐based radiosensitizers have emerged for boosting RT in a targeted and precise manner.^127^ The engineered NPs target the tumors and generate multiple electrons (e.g., Auger electrons, photoelectrons) upon receiving radiation.^114^ Therefore, radiosensitizers can allow the use of lower total radiation doses to induce tumor destruction while preserving the adjacent healthy tissues.

The combination of two or more separate therapeutic modalities can be an additive or even a synergistic approach to treat cancer, by targeting signaling pathways and altering the tumor microenvironment as well as killing the cancer cells.^4^, ^204^ The cost–benefit balance of combination therapies demonstrates a lower cost and higher benefit of FDA‐approved approaches leading to increased efficacy.^4^ For instance the use of immune checkpoint inhibitors for immunotherapy can improve the outcomes of surgery and chemotherapy as well as RT.^205^

The release of danger‐associated molecular patterns (DAMPs) characteristic of immunogenic cell death (ICD) caused by RT, increases the immune response via attracting and maturing dendritic cells, and increasing CD8+ T cell infiltration within the tumor.^206^ Mild hyperthermia synergizes with immunotherapy by increasing the release of heat shock proteins (HSPs), as well as the release of tumor antigen‐containing exosomes, and increasing immune cell infiltration via vascular dilation.^207^ On the other hand, the release of DAMPS following the ablative effect of high‐temperature hyperthermia, or after PDT can provide a similar boost to the immune response as RT.^208^, ^209^ Utilizing drug delivery approaches alongside RT has shown promise to treat cancer while minimizing the RT side effects.^210^ In addition, nitric oxide delivery is a recently emerging approach in cancer therapy, which enhances the RT effect in hypoxic tumors via angiogenesis regulation^211^ and a direct anticancer effect.^212^ The synergistic effects of radiosensitizing NPs have been studied in combination with several approaches discussed below.

### 5.1 Immunotherapy

The combination of RT and immunotherapy has recently been reviewed,^213^ while this idea dates back to the clinical observation of raised antibody levels and T‐cell markers following RT.^214^, ^215^ Next RT was found to sensitize tumors to respond to immunotherapeutic drugs leading to an overall increase in the efficacy of the therapy.^216^ Combination therapy of NBTXR3 and immunotherapy is under phase III of clinical trials using cetuximab on locally advanced head and neck squamous cell carcinoma^217^ and phase I using Nivolumab/ Pembrolizumab on several advanced cancers.^217^

The investigation of cell death mechanisms caused by RT has unveiled their role in the immune response. Cancer cells undergo immunogenic cell death (ICD) and release DAMPs, neoantigens, and cytokines, which then direct the antigen presenting dendritic cells to undergo maturation and travel to the draining lymph nodes.^218^, ^219^ For instance, high mobility group box‐1 protein (HMGB‐1) is a DAMP which contributes to DC activation by binding to TLR4.^220^ Furthermore, ATP release following RT acts as a pro‐inflammatory factor for activation of DC inflammasomes.^221^, ^222^ Beyond ICD induction, radiation monotherapy is not able to elicit an effective immune response.^223^, ^224^

Major routes for immunosuppression include the programmed cell death protein 1 (PD‐1) and its two ligands (PD‐L1 and PD‐L2). Immunosuppressive mechanisms governing T cells are inhibited in checkpoint blockade immunotherapy to stimulate systemically anticancer immune responses. Effector T cell function in tumors is suppressed by the binding of PD‐1 with either of its ligands, which suppresses the kinase signaling pathways involved in T cell activation. Metal–organic framework (MOF), made from ion or clusters of nanomaterials interconnected via organic linkers, shows promising results in the delivery of drug/antibodies^225^, ^226^ and radiosensitizing activity.^227^

Ni et al.^228^ fabricated a hafnium‐based MOF which allowed superior generation of hydroxyl radicals (•OH) in comparison with hafnium oxide NPs. The combination of the NPs and an anti‐PD‐L1 antibody increased the CD4+ and CD8+ T‐cell ratio, and caused immunologic cell death, along with calreticulin expression and neoantigen release. Moreover, researchers also used two‐dimensional nanosheets (1.6 nm thickness) containing the hafnium MOF.^229^ The MOF structures constructed via porphyrins (hafnium‐5,15‐di(p‐benzoato) porphyrin, 5,10,15,20‐tetra (p‐benzoato) porphyrin, and etc.), produces singlet oxygens in addition to •OH, demanding a lower radiation dose for inducing abscopal effect.^230^

Dong et al.^231^ described a radiosensitizer which also allowed a photothermal effect in the form of a WO2.9‐WSe2‐PEG heterojunction. The nanoparticles plus anti‐PD‐L1 co‐administration elicited cytotoxic T‐cells and an anti‐tumor memory effect with a low dose of RT. The radioimmunological performance of nanoparticles may modulate the immune microenvironment of remotely grown tumors by abscopal effect.

Zhang et al.^232^ used radiotherapy‐activated NBTXR3 NPs for an abscopal anti‐tumor effect in mice with colorectal cancer. This study results showed that the use of radiotherapy plus NBTXR3 NPs could increase the necrosis and apoptosis of cancer cells compared with radiotherapy alone.^232^ Indeed, the increased number CD8+ cells and remote regulation of immunologic gene expression of the secondary tumors under abscopal effect is correlated with tumor volume reduction.^233^, ^234^ Also, NBTXR3 activates the cGAS‐STING pathway which facilitates the IRF3/7 transcription factor entrance to the nucleus and type‐1 interferon (IFN‐1) release thereof.^131^, ^235^ As higher dose of radiation may trigger cytosolic DNA degradation via TREX‐1 exonuclease, using a proper radiation dose is demanded in order to leverage STING pathway mediated immunotherapy.^236^

In order to enhance the radioimmunotherapy response, utilizing the capability of polymeric based nanoparticles is addressed. Patel et al.^237^ used bacterial membrane coated polyplex nanoparticles containing PC7A (pH‐responsive polymer) and CpG (TLR9 agonist). In these nanoparticles, the released neoantigens following RT were trapped by maleimide modification of the NPs and transported toward dendritic cells for the next immunologic steps. The maleimide functional groups captured the neoantigens by forming thioester bonds.^238^ In a study by Pang et al.^239^ polysaccharide NPs extracted from *Astragalus membranaceus* natural herb were used to inherently activate DCs. These nanoparticles inhibited both primary and secondary tumor growth in combination with RT, which agreed with the increased populations of CD4+ and CD8+ T‐cells. These advantages led to a robust antitumor immune response in combination with checkpoint blockade inhibitors.^228^

Chen et al.^240^ encapsulated the enzyme catalase and R837 (imiquimod, TLR7 agonist) within PLGA nanoparticles. Catalase serves as a catalyst to transform hydrogen peroxide into molecular oxygen, which is required for RT to be fully effective. In this study, the co‐administration of nanoparticles and anti‐CTLA4 checkpoint blockade led to suppression of Tregs, which potentiated the immune response against metastatic tumors. Moreover, smart radiation‐responsive NPs, which contained a radiation responsive linkage, have been synthesized to allow a triple combination of RT, immunotherapy, and chemotherapy.^241^, ^242^

### 5.2 Gene and nucleic acid delivery

Combining gene therapy approaches with RT may be a promising strategy to overcome tumor resistance to radiation, and increase the therapeutic response to RT.^243^ The use of radiation inducible promoters for genes involved in cell cycle checkpoints, cellular stress, DNA repair, and apoptosis could lead to more effective and specific RT, and reduce off‐target effects.^243^ Kaliberov et al.^243^ have extensively reviewed the combination of RT with the delivery of various transgenes, including gene therapy strategies responsible for DNA repair pathways (e.g., BRCA1 and BRCA2), sensitizing tumor cells to radiation (e.g., high‐affinity membrane receptors), modulation of apoptosis pathways (e.g., P53, Bcl‐2, Bcl‐xl, and survivin), targeting the tumor microenvironment (e.g., VEGF, EGFR, TNF‐α), immunomodulation (e.g., various cytokines), and oncolytic virotherapy. On the other hand, different NPs have been used for more effective gene delivery for cancer treatment, as discussed elsewhere.^244^

Given the complex physiology of tumor cells and their various tumor escape mechanisms, combination therapies often result in better responses. However, using several therapeutic approaches together could also increase the treatment side effects.^243^, ^245^ As mentioned before, specific approaches could be used to improve the sensitivity of tumor cells to RT. Yang et al.^246^ have designed zwitterionic Au‐containing dendrimers to deliver hypoxia inducible factor‐1α (HIF‐1α) silencing siRNA while providing radio‐enhancing effect. Knocked‐down HIF‐1α plus radiation have decreased the metastatic behavior through downregulation of vascular endothelial growth factor (VEGF) and matrix metalloproteinase 9 (MMP‐9) expression. Although radiosensitizing NPs and gene delivery have been used separately for sensitizing tumor cells to radiation therapy,^243^, ^245^ however, the publications on their combination are scarce. Undoubtedly, targeting multiple pathways with gene therapy approaches coupled with radiosensitizing NPs could be a promising future strategy.

### 5.3 Photothermal therapy (PTT)

The success of radiosensitization approaches is highly dependent on the type of tumor that is targeted. There are three primary types of DNA lesions involved in RT: SSB, DSB, and damage to nucleic acid bases.^247^ SSB, the breakage of one DNA strand, is seen when one Gray of radiation elicits about 1000 SSB. However, SSBs are ultimately repaired through cellular processes leading to low efficacy of cell death. On the contrary, DSBs where both complementary strands of the DNA backbone are broken is considered to be preferable due to the less efficient cellular repair. DSBs result in much higher cell death and mutations. However, the limitation is the low incidence of this type of breakage, only of the order of 40 DSB for each Gray of radiation. The last‐mentioned type, local nucleic acid base damage, is associated with the concurrent occurrence of DSBs and SSBs, with only a few bases affected. Meanwhile, alteration of cell‐signaling pathways by hyperthermia can lead to the impairment of DSB repair processes. Hyperthermia sensitizes the cancer cells to various treatments, such as chemotherapy^248^ and RT.^249^ For instance, hyperthermia could increase the uptake of platinum‐based drugs by colorectal cancer cells, and produced more G2 cell cycle arrest and cell death.^250^

The effect of hyperthermia is highly dependent on the phase of the cell cycle. Accordingly, cells in the M and S phases of the cell cycle are more vulnerable toward hyperthermia.^251^ In contrast, the G phase is resistant to heat treatment, which should be taken into account because radiation is more effective with cells in the G2/M phase.^252^

The poorly developed vasculature of the tumor leads to an oxygen‐deprived and acidic microenvironment, which diminishes the efficacy of RT. Hyperthermia at high temperatures directly kills the cells, even within a hypoxic environment through unclear mechanisms.^253^, ^254^ However, direct cell killing at low temperatures under hypoxic conditions requires a more prolonged cycle of hyperthermia.^255^ In addition, cells treated with hyperthermia express HIF‐1α, which switches the cellular metabolism into glycolysis, leading to diminished oxygen consumption.^256^, ^257^

Moreover, hyperthermia has been shown to be promising in some clinical trials to enhance the efficacy of RT.^258^, ^259^ The effect of in vivo hyperthermia at mild temperatures (40–42°C) may lead to vasodilation, and therefore greater oxygen delivery to the tumor, thus sensitizing the cells to RT.^260^ Drainage of the interstitial fluid under elevated temperature is considered proof of increased oxygenation.^261^ Obtaining uniform distribution of the heat over the entire heterogeneous structure of the tumor is the main hurdle against hyperthermia‐mediated therapy.^262^ Progress in nanomedicine and the heat transfer of fluids could offer novel tools to solve this issue. Moreover, cells are more vulnerable to death from NP‐mediated hyperthermia than other more conventional heat generation methods.^263^

Regarding the physical processes for heat generation by NPs, they can be divided into two main subgroups, PTT and magnetic NPs excited by an alternating magnetic field. The photothermal effect in NPs is governed by two mechanisms, localized surface plasmon resonance (LSPR) and non‐radiative relaxation. LSPR occurs following the matching of the wavelength of the absorbed photons to the frequency of the oscillating surface electrons under the control of the positively charged nuclei at the atomic scale.^264^ Gold NPs can be synthesized in various morphologies, which govern their highly size‐dependent and shape‐dependent interactions with light.^264^, ^265^ These characteristics have attracted researchers to use gold NPs in therapy, imaging, and biosensing applications.

The other mechanism of photothermal generation arises from the semiconducting properties of defined NPs. The bandgap between the conduction and valence electrons of the semiconducting materials governs their photon absorption and the following events. In other words, incident photons can provide the energy for the electrons in the valence band to reach the conduction band. Return of the excited electrons in the conduction band to the ground state is accompanied by heat generation by a process called lattice‐phonon interaction. In contrast, magnetic (especially superparamagnetic) NPs produce heat upon stimulation with an alternating magnetic field, which has been fully discussed elsewhere.^266^

Table 3 summarizes some studies on the combination of PTT and NP radiosensitizers. A study by Li et al.^267^ showed that PTT prior to RT with RGD‐modified gold NPs markedly increased the number of cells in the G2/M phase, producing high levels of apoptosis. PTT agents are commonly designed to absorb light within the NIR‐I region by adjusting the shapes and composition. Meanwhile, hybrid materials can be synthesized, which can carry out therapy and imaging, in a theranostics approach.

**TABLE 3:** Combination of PTT and radiosensitization mediated by nanoparticles.

Morphology	Nanoparticles	Size (method)	Radiation	Dosage (Gy)	Laser wavelength (nm)	Power (W/cm^2^)	Duration (min)	In vitro	In vivo	Key finding	References
0D	MoS_2_ QDs@PANI‐PEG	21.5 ± 3.9 (TEM)	X‐ray	6	808	1.5	5	4 T1	Balb/c nude mice	Increased temperature from 25 to 54.8°C induced an ablative response	403
	BSA‐Bi	39.52 (DLS)	X‐ray	4	808	0.75	5	4 T1	Balb/c mice	Photothermal conversion of 51%	404
	FePd‐Cys	≈3.4 (TEM)		6	1064	1	–	4 T1	Balb/c mice	Hypoxic tumor tissue after PTT (14.3%) was less than control (71.5%)	405
	Au‐folate	5–20 (TEM)	6‐MV	2	532	0.47	15	KB	–	Increased apoptosis after combination therapy	406
	Cs‐Au‐ICG	Au: 5 nm (TEM) Cs‐Au‐ICG: 53.1 ± 11.7 (TEM)	–	6	808	1.5	–	Hep‐l, 293 T, 4 T1	Balb/c	Assembly by pH increased tumor accumulation; disassembly by NIR laser produced ablation, Decreased liver and lung metastasis	407
	AuPd‐PVP	≈30 (TEM)	–	5	808	0.5	–	MC38	Mice	Apoptosis was produced Massive PTT effect in‐vivo	408
	Mesoporous Pt	≈320 (TEM)	6 MV, 200 cGy/min	6	808	1, 1.5		C540 (B16/F10)	–	Increased cytotoxicity with combination therapy	409
	Pt	12.2 ± 0.7 (FESEM)	6 MV, 200 cGy/min	6	808	1.5	–	C540 (B16/F10)	–	Combination therapy effect higher at 72 h than 24 h	410
	Fe_3_O_4_@Au/Alg	~78.8 (DLS)	6 MV, 200 cGy/min	2, 6	808	1.0	–	KB	–	Cell cycle arrest at G2/M phase at 24 h post laser irradiation	411
	Gd_2_O_3_/BSA@MoS_2_‐HA	Gd_2_O_3_ ~ 4 (TEM) Gd_2_O_3_/BSA@MoS2‐HA ~190 (TEM)	–	6	808	1.5	–	4 T1	Kunming mice	Good tumor uptake; facile removal from blood circulation	412
	MnO_2_‐mSiO_2_@Au	≈142.5 (TEM)	–	6	808	1.5	5	4 T1	Kunming nude mice	H_2_O_2_ converted to molecular oxygen by Mn2+; increased radiation‐induced DSBs	298
	Polyvinylpyrrolidone‐ Bi_2_Se_3_@Selenocysteine	15 ± 3 (TEM)	–	6	808	1.0	10	BEL‐7402	BALB/c nude mice	Selenium released into blood decreased toxicity of RT; improved immune function	413
	Cu_2_‐xSe‐Au‐PEG	37.9 (DLS)		6	808	0.75	9	4 T1	BALB/c mice	Enhanced PTT conversion and X‐ray sensitization by heterostructure design	414
	Iodine[131I] doped‐CuS‐PEG	≈20 (TEM)	–	–	808	0.25	25	4 T1	Balb/c mice	Radioactive NPs with PTT ability provided 85% survival versus 0% in controls	415
	BiOI@Bi_2_S_3_@BSA	≈100 (TEM)	–	6	808	1	10	BEL‐7402	Balb/c nude mice	Heterostructure configuration produced high levels of ROS	416
	Lutetium‐177/Au‐RGD‐NLS‐Aptamer	≈20 (TEM)			532	1.19	3.5	U87MG	Athymic male mice	Cell viability and tumors reduced by combination therapy	417
	Mesoporous polydopamine sponge‐WS_2_@MnO_2_	≈170 (TEM)	X‐ray	6	808	1.5	5	4 T1	Mice	MnO_2_ catalyzed the conversion of H_2_O_2_ to O_2_	297
	WS_2_	3(TEM)	X‐ray	6	808	1	10	4 T1	Balb/c nude mice	γ‐H2AX increased 2.1fold by combination therapy	418
	Au@Fe_2_O_3_	≈50 (TEM)	6 MV, X‐ray	2	808	6	10	KB	‐	Apoptosis ratio increased versus necrosis	419
	Au@Fe_2_O_3_	20–60 (DLS)	6 MV, X‐ray	2, 4	808	2	5	KB	–	Apoptosis (Bax/Bcl2 increased)	420
	Mesoporous silica/CuS/red blood cell (RBC) content/RBC membrane	109 ± 10 (TEM)	X‐ray	4	980	1	10	4 T1	–	Increased RBC transport of O_2_; increased cell death	421
	Fe_3_O_4_@Au	100 (DLS)	X‐ray	5	808	15	5	HeLa	–	Decreased cell survival from 74.3% to 40.2% with X‐ray combination Magnetic field increased cell uptake & cell death	422
	Folic acid‐PEG‐MoSe_2_@BSA	139.8 (TEM)	X‐ray, 0.084 Gy/s	5	808	1	5	4 T1	Balb/c nude mice	92.8% of cell death in combination	423
1D	Bi_2_S_3_‐platelete membrane	L 100, D 15 (TEM)	1.0 Gy/min	5	808	1	–	4 T1	Balb/c mice	S phase decreased & G2/M phase increased after PTT	424
	Bi_2_S_3_	155.4 ± 2.8 (DLS)	1.23 Gy/min	4	808	2	–	4 T1	Balb/c mice	Angiogenic factors downregulated; HIF‐1α decreased due to oxygenation post PTT	425
	Au‐Cancer cell membrane	L 68 ± 5 D 11 ± 1 (TEM)	–	4	980	0.5	1	KB	Balb/c nude mice	Selectively targeted KB cells	426
	Au ‐ RGD	L 44.4 D 15.6 (TEM)	6 MV, X‐ray	4	808	1	60	A375	–	Increased apoptosis	267
	Cu_3_BiS_3_	L 25 D 8 (TEM)	X‐ray	8	1064	1	6	4 T1	Balb/c nude mice	40.7% PTT conversion at NIR‐II region	270
	Bi_2_S_3_ ‐ Au	34.5 (TEM)	X‐ray	6	808	0.3	10	4 T1, HeLa	Balb/c nude mice	Schottky type heterostructure decomposed cellular H_2_O_2_ to HO•	427
	Pt	100 (DLS)	X‐ray	6	1064	0.75	5	4 T1	Balb/c mice	Conversion efficiency of 44.7%	269
	W doped TiO_2_	L 9.1 ± 2.2 D 5.7 ± 1.4 (TEM)	X‐ray	4	1064	1	10	4 T1	Balb/c mice	Conversion efficiency of 44.8%	428
2D	Silicene@Pt	200 (DLS)	γ‐ray	6	808	1.25	–	4 T1	Balb/c nude mice	Decorated Pt served as radiosensitizer/CT contrast agent & catalyst for converting H_2_O_2_ to O_2_	429
	rGO/Au@Fe_3_O_4_	29.4 (TEM)	6 MV	2, 4	808	1.8	5	KB	–	PTT decreased viability from 49.8% to 11.9% at 4 Gy	430
	Titanium disulfide‐HAS‐FA	135.3 (DLS)	–	5	808	0.8	5	CT26	Mice	FA modification and PTT/RT gave cell viability (≈54% versus ≈ 23%)	431
	rGO‐PEG	289 ± 4 (DLS)	120 kV, 22.7 mA	10	960	2	60	B16F10	–	Reduced GO increased absorption at 960 nm and PTT	432
	Iodine[131I]‐rGO‐PEG	≈50 (AFM)	–	–	808	0.2–0.3	20	4 T1	Balb/c mice	131I‐rGO alone and with light irradiation showed more cell death than radionuclide	433
	Pd@Au	30(TEM)	X‐ray	8	1064	0.3	–	4 T1	–	Nanoparticle catalyzed H_2_O_2_ to O_2_	421
3D	BiP_5_W_30_/rGO	–	50 kV, 75 μA	6	808	1	–	HeLa	Balb/c nude mice	Heterostructure configuration increased ROS generation GSH levels were diminished	351
	Bi_2_S_3_‐S‐S‐HA/GA	40	–	6	808	0.5	–	4 T1	Balb/c nude mice	Redox responsive GA release	434
	Gold	150 (TEM)	–	6	808	1	–	PANC‐1 SW1990	Balb/c nude mice	SW1990 tumor size was reduced by 96.6% Increased oxygenation was shown imaging	435
	Gold‐PEG‐CD44 antibody	58.14 ± 4 (TEM)	6MV	4	808	2.5	5	4 T1	Balb/c mice	Antibody‐targeted radiosensitization	436
	Gd‐Polytungstate	≈3.5 (DLS)	–	6	808	1	10	BEL7402	Balb/c mice	Polyoxo‐tungstate decreased cell viability, showed low toxicity	437
	Mn/Hf‐IR825‐PDA‐PEG	≈100 (TEM)	–	6	808	0.3	20	4 T1	Balb/c mice	Co‐doping with Mn2+ and Hf + allowed PTT, RT and PA/CT imaging	438
	Prussian blue@Au	≈138.8 (TEM)	6 MV, X‐ray	4	807	1.5	5	4 T1	Balb/c mice	Integration of Prussian blue NPs and AuNPs allowed MRI/CT imaging and PTT/RT	439
	Eutectic gallium indium‐Au	≈200 (TEM)	–	6	808	1.5	10	4 T1	Balb/c mice	PTT conversion efficiency was low; needed higher concentration of NPs	440
	Au@Pt	30 (TEM)	X‐ray	4	808	1	10	4 T1	–	Cell viability was 32% with combination; High absorption at long wavelengths	441
	Au	≈70 (DLS)	–	5	1064	2	10	EMT‐6	Balb/c mice	Hypoxia was reduced after mild hyperthermia; vascular dilation	271
	Au ‐ PEG	54 ± 9 (TEM)	6 MV, X‐ray	4	808	2	5	KB	–	Higher DNA damage with combination	442
	PtCu‐PEG‐FA	64.2 (TEM)	X‐ray, 120 kvp	10	808	2.4	5	HepG_2_	Nude mice	Tumor was eradicated at 18 days; Negligible difference between 778, 808 and 980 nm lasers	443

**FIGURE 7:**
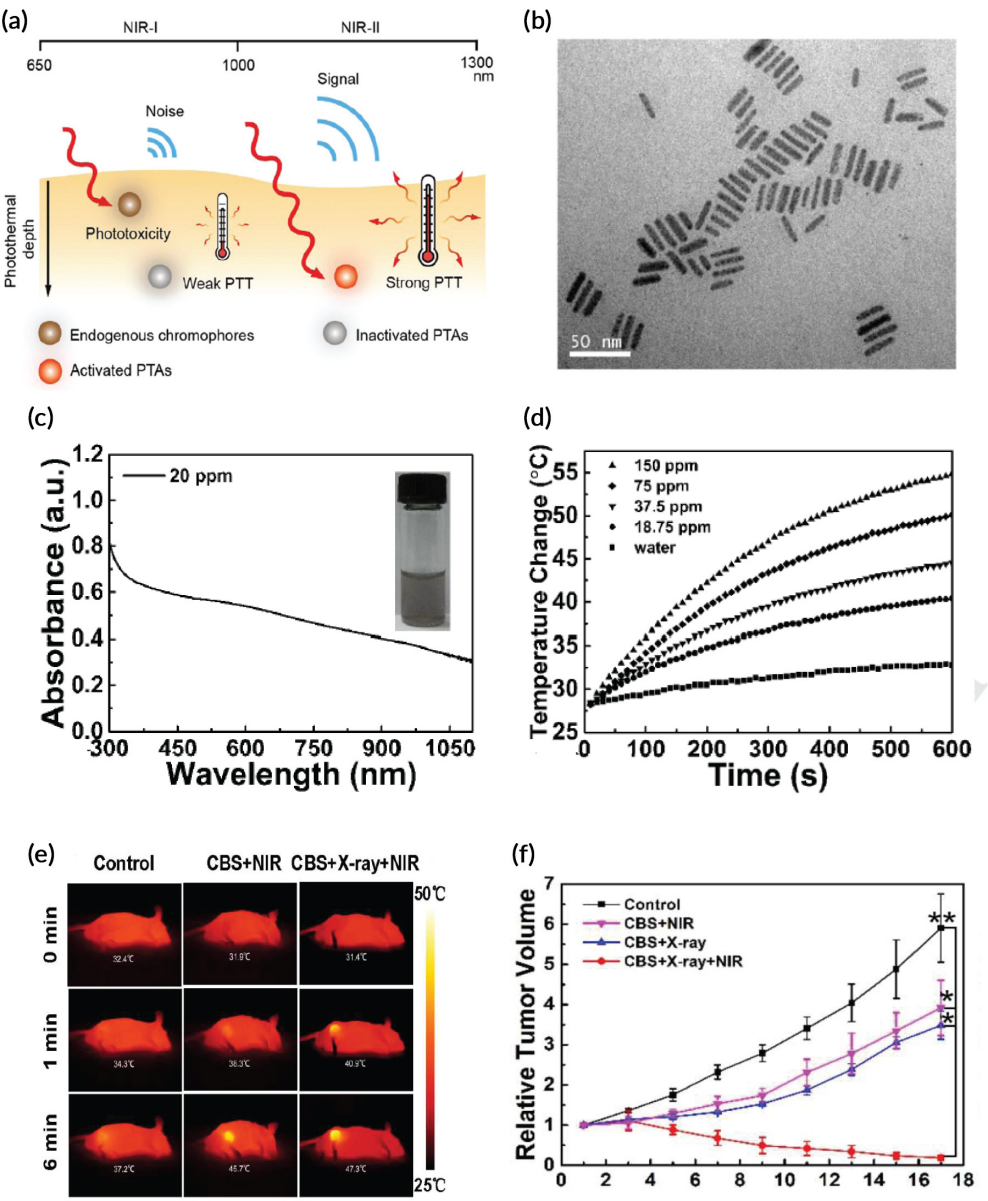
Radiosensitization with Cu3BiS3 nanorods. (a) Schematic of PTT in NIR‐I versus NIR‐II region; (b) TEM images of the Cu3BiS3 nanorods; (c) UV‐VIS spectra of the Cu3BiS3 nanoparticles; (d) photothermal effect of the nanoparticles at various concentrations; (e) Temperature change by PTT and PTT + X‐ray therapy of CBS; (f) tumor size changes with PTT, X‐ray, and PTT + X‐ray of CBS treated 4T1 tumors, reprinted with permission from Reference 282

Moreover, light in the near infrared‐II (NIR‐II) region has lower scattering/absorption by tissue constituents compared with NIR‐I, allowing deeper tissue penetration for PTT applications as schematically shown in Figure 7a.^268^ In a study by Ma et al.,^269^ platinum nanoworms located under 0.9 mm of pork tissue showed 44.7% and 28.1% photothermal conversion efficiency at 1064 nm and 808 nm wavelengths, respectively. Li et al.^270^ synthesized copper bismuth sulfide (CBS Cu_3_BiS_3_) nanorods, a ternary semiconductor nanoparticle, with evenly distributed length and diameter as shown in Figure 7b. In Figure 7c, the absorption spectra in the NIR‐II region (1000–1100 nm) coupled with the semiconducting properties provided a high photothermal conversion efficiency of up to 40.7%. The photothermal effect in both in vitro and in vivo conditions caused 4 T1 tumor volume reduction within 17 days, as shown in Figures 7d–f.

NIR‐II PTT in combination with radiosensitization was reported by Li et al.^271^ using penta‐twin‐shaped gold NPs. These NPs showed a high extinction coefficient and gave a 44.2% photothermal conversion efficiency. Mild hyperthermia caused the dilation of tumor vasculature and increased oxygenation, leading to an increase in radiosensitization.

### 5.4 Photodynamic therapy

Photodynamic therapy (PDT) is an emerging technology for treating cancer, with some clinical approvals in recent decades. In this method, the light is aborbed by a photosensitizer (PS) and initiates a photochemical reaction leading to ROS generation by two mechanisms, types I and II. In type 1, electrons are transferred from the excited triplet state PS to adjacent biomolecules to form radical cations and radical anions, that go on to form ROS.^272^ In type 2 PDT the excited PS interacts with surrounding molecular oxygen to form the extremely reactive singlet oxygen.^272^ These ROS oxidizes biomolecules inside cells (lipids, proteins, and nucleic acids) or damages the plasma membrane, resulting in cell death.^273^, ^274^, ^275^ PDT has been shown to deoxygenate and affect the physical properties of the tumor microenvironment.^276^ Porphyrins, a common type of PS compound, are found naturally in hemoglobin and chlorophyll. Porphyrins are made up of four mono‐pyrrole rings, which are connected in a macrocycle via methine bridges.

The penetration of light to deep tissues is restricted due to the scattering by tissues and absorption by hemoglobin. However, NIR‐I (700–1000) and NIR‐II (1000–1150) light wavelengths have the lowest scattering and absorption when passing through tissue.^277^ Although the NIR‐II window has the best tissue penetration, it suffers from insufficient photon energy for the excitation of electrons to the upper molecular orbital. This issue could be addressed if X‐radiation could be utilized as a photon‐based energy source. Following this approach, several types of X‐ray‐mediated photosensitizers (XMP) have been reported. Generally, XMPs can be categorized into two groups, PS decorated agents and high‐Z NPs.

PS molecules can be anchored onto the surface of common radiosensitizer NPs to generate additional ROS. In addition, the combination of PS and scintillation elements is another method in which ROS are produced. In this technique, the scintillation elements are excited to emit light upon absorbing X‐rays. The scintillation material can supply the photons required for PDT for a period of time after the X‐rays have finished. Therefore, the toxicity of prolonged X‐ray irradiation is decreased while higher throughput can be achieved.

Hetero‐structured hybrid NPs have recently gained attention for deep XMP of the tumors. In this method, two attached semiconductors with distinct band gaps yield ROS through a photochemical reaction. The use of a high‐z element as a semiconducting component of this structure is prioritized owing to their higher X‐ray attenuation, and direct electron (Auger) generation.^278^

### 5.5 Oxygen delivery

Oxygen is an essential component of RT, and increases the overall efficacy by improving ROS generation within the tumor. The earlier drugs used for improving the oxygen level of the tumor microenvironment were discussed previously. Oxygenation of the tissue using biomaterials, is often carried out by loading molecular oxygen into rationally designed formulations, which then produce free oxygen following dissociation.

Perfluorocarbon (PFC)‐based materials have been clinically used as artificial blood substitutes, due to their high gas dissolving capacity and chemical inertness.^279^ Certain compounds (sodium percarbonate, hydrogen peroxide, and calcium peroxide^280^) can generate oxygen when they come into contact with water, or are catalyzed by catalase. Beyond the delivery of physically entrapped molecular oxygen, NPs with inherent oxygen generation capability can operate by water splitting, or by converting tumor‐residing hydrogen peroxide into oxygen.^281^

**FIGURE 8:**
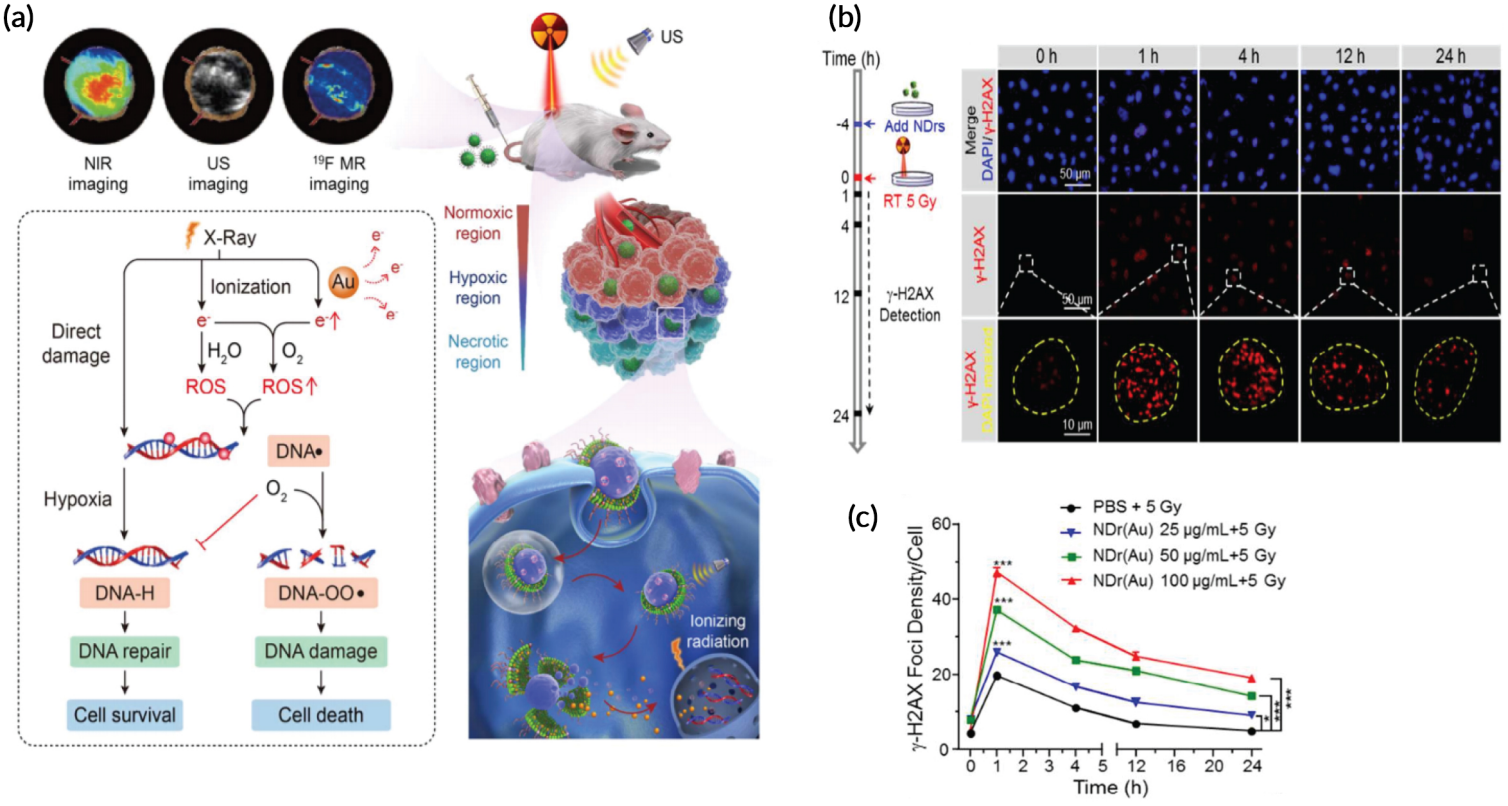
Radiosensitization with gold nanodroplets. (a) Schematic illustration of hierarchical multiplexed nanodroplets and mechanism of action; (b) qualitative; and (c) quantitative content of γ‐H2AX after 5 Gy of X‐ray radiation, reprinted with permission from Reference 297

NPs can be incorporated with PFCs to deliver more oxygen for sensitizing tumors to RT.^282^ Song et al.^283^ fabricated PEG‐modified hollow Bi_2_Se_3_ NPs, which served as a reservoir for PFC delivery and thermo‐radiation therapy of the tumor. Active oxygen delivery was demonstrated by an accelerated rate of oxygen release rate upon laser irradiation. The same group reported a higher PFC loading, using a PFC nanodroplet hybrid with high‐Z tantalum oxide (TaOx) (TaOx@PFC‐PEG) nanoparticles. This system was able to considerably increase RT efficacy owing to its multifunctional activity.^284^ Ultrasound‐triggered oxygen release was reported by Jiang et al.^285^ using hierarchical multiplexed nanodroplets of liquid perfluorooctyl bromide and ultrasmall gold NPs as an oxygen carrier and radiosensitizer, respectively. The NPs were able to prevent unwanted DNA DSB repair by delivering oxygen to the cells, as shown in Figure 8a. To prove the concept, the γ‐H_2_AX content (molecular marker of DNA damage and repair) was measured at different time intervals post‐X‐ray irradiation (5 Gy) (Figure 8b, c).

Other method for oxygenating the tumors is to utilize the inherently accessible hydrogen peroxide and convert it to the molecular oxygen through catalytic reaction. Therefore, NPs bearing catalase‐like activity could be leveraged for oxygen‐feeding. Namely, Porphyrin‐based MOFs decorated with gold nano‐radiosensitizer and hafnium‐based manganoporphyrins increased the oxygen level up to 6 and 23 mg/L, respectively, in presence of H_2_O_2_.^225^, ^286^ Cyanobacteria are considered as the initial oxygen generator on the earth utilizing the following reaction^287^:

(1) $$6\;{\mathrm{CO}}_{2}+6\;H_{2}O+\text{energy}\left(\text{photon}\right)=C_{6}H_{12}O_{6}+6\;O_{2}$$

Different strains of cyanobacteria are able to produce nutrients and remove the unwanted compounds from the medium.^288^ Amongst with, *Synechococcus elongatus* has been used in vitro and in vivo for supplying the molecular oxygen for ischemia^289^, ^290^ and PDT.^291^, ^292^ Chai et al.^293^ has co‐injected the *Synechococcus elongatus* cyanobacteria (activated by 660 nm light) and bismuthine (4Gy), able to control the tumor's volume and weight within 2 weeks post irradiation. *Synechococcus elongatus* has micrometer size which would restrict its accumulation in tumor site through EPR effect.^293^, ^294^ Also, under radiation with high energy, the DNA of the cyanobacteria is attacked by ROS and it's O_2_ evolution is disrupted.^295^

MnO_2_ can be reduced to Mn^2+^, and its reaction with tumoral hydrogen peroxide produces copious amounts of oxygen. Fan et al.^296^ synthesized up‐conversion NPs decorated with MnO_2_ nanosheets for laser and X‐ray mediated PDT/RT. In addition, the up‐conversion NPs allowed H_2_O_2_/pH dual responsive luminescent bioimaging when exposed to an NIR laser. Hybrids of MnO_2_ have been used in combination therapy, as summarized in Table 2.^297^, ^298^ Using a similar mechanism, mesoporous platinum was used for catalytic oxygen generation plus radiosensitization.^299^

### 5.6 Drug delivery

For decades, many drugs have been designed to kill cancer cells by distinct mechanisms, and nowadays, rationally designed drug delivery platforms have gained much attention to decrease the toxic effects of systemic drug administration. Progress in drug discovery has led to the production of several radiosensitizing drugs to boost radiotherapy.^300^, ^301^ Most recently, Yi et al.^302^ reviewed the concept of NP design for the delivery of drugs and radiosensitizer agents, and concentrated on drug‐radiosensitizer combination effects.

Recently, the combined delivery of NP‐based radiosensitizers and drugs (also known as chemo‐radiotherapy) has been shown to increase DSB formation by various mechanisms^303^ (Table 4). Bannister et al.^304^ investigated the role of docetaxel in stabilizing microtubules in cancer cells (HeLa and MDA‐MB‐231) and preventing the exocytosis of gold nanoparticles (GNPs). Docetaxel directed the GNPs to reside closer to the nucleus, making the cells more vulnerable to RT. Moreover, in some cases synergistic combination approaches can compensate for the inability of RT to induce sufficient DSBs.^305^

**TABLE 4:** Combination effects of radiosensitizers and drug delivery in cancer treatment.

NPs	Surface	Drug	Size (nm)	Cell/Animal	Radiation	Assays	References
Gold	Citrate	Suberoylanilide hydroxamic acid	10	A549 DU‐145 PC‐3	0, 2, 4 Gy	MTT, Colony formation, γ2AX	444
Gold	Folic acid	17‐Allylamino‐17‐demethoxy‐geldanamycin (17‐AAG)	–	HCT‐116	2 Gy, 6 MV X‐ray	Caspase 3 expression	445
Gold	2,3‐Dimethyl maleic anhydride (DMMA)/Polyallyamine(PAH)	Cisplatin prodrug	78.3	B16 C57/BL6 mouse melanoma	0, 2, 4, 6, 8 Gy	Aggregated NPs enhanced tumor retention	446
PLGA	PEG/Folate receptor/Yttrium 90	Paclitaxel	75 ± 10	SW 626, SKOV‐3, OVCAR‐3 Female Nu/Nu mice bearing SKOV‐3 tumors	–	SW626 have lower cell uptake & cell death due to lack of folate receptors	447
Gold	PEG	Cisplatin	50 (Gold core)	S2	1, 10 Gy, Gamma ray	Caspase 3 expression	448
Gold	‐	Bleomycin	1.9	MDA‐MB‐231	6 MV (3.55 Gy/min), 15 MV (3.85 Gy/min)	Increased radiosensitizer enhancement	305
GNRs, GNPs	Dopamine/PEG/RGD	Cisplatin	GNRs (D 22.41 ± 1.01, L 56.12 ± 3.22), GNPs (56.37 ± 3.04)	H1299 Balb/c nude mice bearing H1299 tumors	320 Kv, 6 Gy	GNRs showed rapid accumulation in tumor	449
GNPs	PEG/RGD	Cisplatin	17.2 ± 5.6	HeLa, MDA‐MB‐231	2 Gy, 6 MV	Guided NPs to the nucleus; Cell division inhibited	304
GNPs	RGD	Cisplatin	10	MDA‐MB‐231	2 Gy, 6 MV	No adverse interaction between drug and NPs	303
GNPs	–	Tirapazamine	16.6 ± 2.1	HepG2	50 kVp, 0.5 Gy/min	–	450

### 5.7 Nitric oxide delivery

Nitric oxide (NO) is a naturally occurring free radical gas produced by bacteria and mammals, which plays a significant role in many cellular signaling pathways.^306^, ^307^ NO is synthesized by the transformation of L‐arginine to L‐citrulline by the isoforms of nitric oxide synthase (NOS).^308^ Initial studies on the role of NO in cancer therapy showed its over‐expression in several cancers.^309^ Since then, several studies have investigated its role in cancer and possible ways to fight cancer, in a similar manner to ROS.^310^

According to the levels of NO, its effects are classified as direct reactions (<200 nM) and indirect reactions (>400 nM).^311^ Direct reactions occur immediately upon NO release, which involve direct interactions with biological receptors. Indirect reactions refer to NO reactions with oxygen or superoxide, which then produces reactive nitrogen species (RNS).^312^ Researchers have used NPs to produce intracellular NO in some photoinduced approaches^313^, ^314^, ^315^ and multimodal therapies.^316^, ^317^

NO is a well‐known substance which can increase tissue oxygenation by dilating the tumor blood vessels by several routes.^318^, ^319^, ^320^ In contrast, the use of nitric oxide synthase inhibitors, such as NG‐nitro‐L‐arginine (L‐NNA), can inhibit anti‐apoptotic pathways^321^ and decrease tumor blood flow leading to chronic hypoxia, cancer cell death, as well as protection of normal cells under radiation.^322^, ^323^ Additionally, NO is an important player in the bystander effect, owing to its rapid diffusion across cell membranes. The bystander effect describes the destruction of tumor cells adjacent to (but outside) the irradiated zone by the generation of toxic molecules from the irradiated cancer cells.^324^

In a study by Han et al.^325^ they found that cells that were physically separate from others receiving α‐particle irradiation formed γ‐H_2_AX protein foci as a function of time. Furthermore, NO and RNS can cause mutagenesis by interfering with factors responsible for DNA repair and genome stability.^326^ The bystander effect of NO may lead to the release of superoxide anions following increased mitochondrial permeability and damage.^327^ The addition of NO scavenger molecules into the culture medium diminishes the bystander effects, which confirms the role of NO.^328^

**FIGURE 9:**
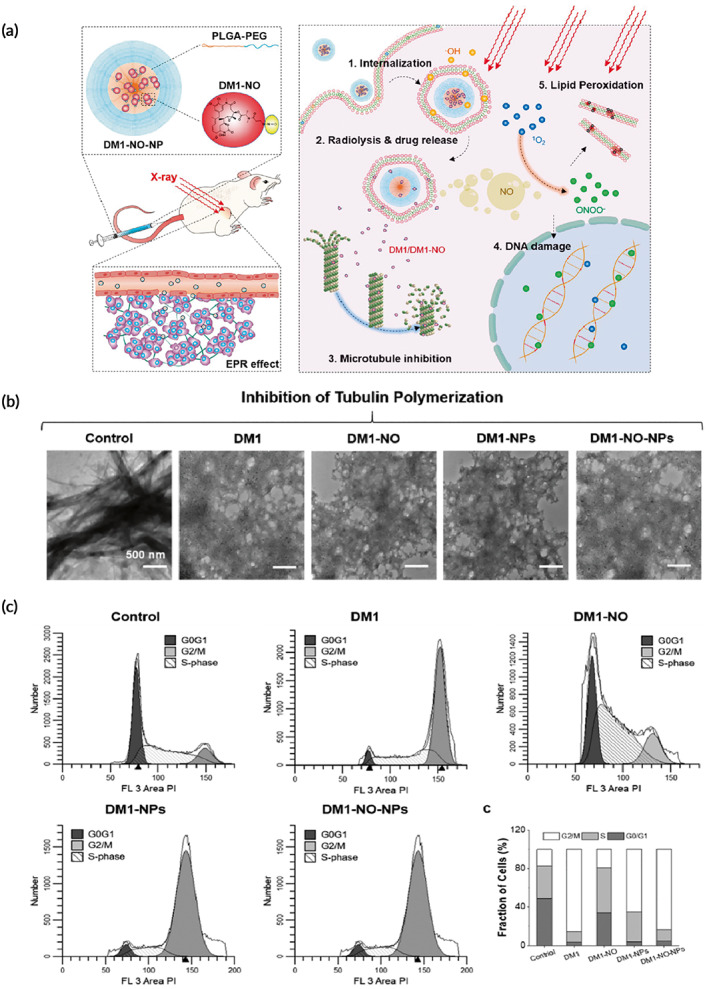
Radiosensitization with NO‐releasing NPs. (a) Schematic of DM1‐NO‐NP synthesis and intracellular effect; (b) TEM images of β‐Tubulin polymerization; and (c) cell phase distribution after nanoparticle RT treatment, reprinted with permission from Reference 342

Researchers have made great efforts to incorporate NO‐producing or NO‐releasing compounds into NP‐based radiosensitizers to increase cancer cell death. Liu et al.^329^ used nitroimidazole as an NO source in a system comprising PEG/cell‐penetrating peptide CPP/GNPs. Nitroimidazole under X‐ray radiation led to production of nitrite ions, which could be directly reduced to NO, or indirectly react with biological cues. Following this approach, Gao et al.^212^ conjugated tert‐butyl nitrite to the maytansinoid DM1 (Figure 9a). Then they encapsulated this conjugate into poly‐(lactic‐co‐glycolic acid)‐block‐poly (ethylene glycol)‐NPs (DM1‐NO‐NPs) (Figure 9b). The designed platform successfully inhibited tubulin polymerization leading to G2/M cell cycle arrest, as shown in Figures 9b, c. Subsequently, 6 Gy of X‐ray radiation increased the superoxide dismutase activity within the cytosol and mitochondria. In this platform, oxidizing agents produced by X‐ray irradiation cleaved the labile S‐N bond, thus creating a smart NO delivery vehicle. These smart approaches can control the NO levels more effectively and diminish the toxicity.^330^ Table 5 summarizes some studies on the combinations of NO delivery and radiosensitization.

**TABLE 5:** Combination of NO delivery and radiosensitization.

NPs	Size (nm)	Coating	Dose (Gy)	Mechanism	Cells/Animal	Effects	Notes	References
LiLuF4:Ce3+/Roussin's black	70	–	6	Peroxynitrite formation from superoxide plus NO	In vitro: A549 cells In vivo: Balb/c mice bearing A549 tumors	DNA damage; PARP inhibition; Tyrosine nitration	Nitration of PARP protein and tyrosine HIF1α decreased; increased tumor oxygenation	331
Bi	36	–	5	S‐nitrosothiol mediated NO delivery, ROS generation	In vitro: HepG2, HeLa In vivo: Zebrafish	DNA damage	Combination of PTT, RT and NO delivery	451
Au/CPP/GNPS	13	PEG, CPP	10	Peroxynitrite formation from superoxide plus NO	A431	CPP targeted the NPs to the nucleus	–	329
PLGA‐co‐PEG‐ maytansinoid DM1 conjugated *tert*‐butyl‐nitrite	78	–	6 Gy, 320 kV	Hydroxyl radical; Singlet oxygen; NO; ONOO‐	In vitro: H1299 Female athymic nude mice bearing H1299 tumor	Mitochondrial and cytosolic SOD increase Tubulin formation inhibited G2/M phase arrest	Normal AST and ALT levels	212
Mesoporous silica‐loaded NaYF4:Yb/Er	137.7	Functionalized with *tert*‐butyl‐nitrite	1, 5, 10 Gy	NO	L929 HeLa cells	–	NO release depends on X‐ray energy	452
Au‐Cluster	100	Platelet membrane‐ PEG	8	Sodium nitroprusside (SNP)	In vitro: CT26, In vivo: BALB/c mice with CT26 xenografts	Hypoxia inducible factor‐1α downregulate, O_2_ consumption decrease	GSH reaction with SNP increased NO level	453

Du et al.^331^ proposed the simultaneous release of NO and superoxide by scintillation nanoparticles constructed of Ce‐doped LiLuF_4_ and Roussin's black (RB) salt (Fe_4_KN_7_O_7_S_3_^+2^) for killing cancer cells. LiLuF_4_ provided the O^2−^ and converted the absorbed X‐rays into UV light, which was necessary for NO production from RB. The reaction between NO and superoxide led to the production of peroxynitrite (ONOO‐), which is more destructive toward DNA, lipids, and lipoproteins than hydroxyl radicals, and is somewhat more stable inside the cells.^331^ This system increased cell death by damaging DNA and inhibiting the expression of PARP protein, a critical DNA repair enzyme.

### 5.8 Fenton reaction‐based therapy

The Fenton reaction catalyzes the conversion of intrinsic hydrogen peroxide within tumors to the highly reactive radicals •OH.^332^, ^333^, ^334^ It should be mentioned that •OH have the highest redox potential (oxidizing capacity) in comparison with other ROS, such as ^1^O_2_ (E(1O_2_/H_2_O) = 2.17 V) and H_2_O_2_ (E(H_2_O_2_/H_2_O) = 1.78 V). Therefore, the toxicity of •OH against cancer cells is superior. Fe_3_O_4_ composes superparamagnetic NPs (SPIONS), which have been widely used as MRI contrast agents. The Fenton reaction on the Fe_3_O_4_ surface or ionic Fe^3+^ released following degradation are responsible for hydroxyl radical generation as shown in these equations:

(2) $${\mathrm{Fe}}^{3+}+O_{2}•{}^{-}\to {\mathrm{Fe}}^{2+}+O_{2}$$

(3) $${\mathrm{Fe}}^{2+}+H_{2}O_{2}\to {\mathrm{Fe}}^{3+}+\mathrm{OH}+•\mathrm{OH}$$

(4) $$O_{2}•{}^{-}+H_{2}O_{2}\to O_{2}+{\mathrm{OH}}^{-}+•\mathrm{OH}$$

Ren et al.^335^ designed a system of mesoporous Prussian blue doped with bismuth sulfide QDs, for inducing oxidative stress while benefiting from the high photothermal activity of bismuth sulfide. Prussian blue acts as a consumer of glutathione and a hydroxyl radical generator by the Fe(II) and Fe(III) incorporated into the nanocarrier.

As previously noted, GSH is the major overexpressed antioxidant in the tumor which corroborates the cancer cells survival.^336^ Using Reaction (4), GSH interaction with Fe^3+^ regenerates Fe^3+^ to the toxic Fe^2+^ consumes it:

(5) $${\mathrm{Fe}}^{3+}+\mathrm{GSH}\to {\mathrm{Fe}}^{2+}+\text{GSSG}\;\left(\text{glutathione disulfide}\right)$$

In other words, the reduction of metallic/semi‐metallic ions (e.g., Cu^2+^) are accompanied by the oxidation of GSH, leading to the GSH depletion. These elements include Mn^2+^, Mo^4+^, Cu^+^, and W^4+^.^337^ Moreover, the increased population of ROS aggravates the GSH depletion.^338^ Loading of the organic compounds such as hemin has endowed a peroxidase‐like activity for removal of the GSH.^339^

Cu^2+^ transformation to Cu1+ shows higher catalytic reaction rate in comparison with Fe^3+^/Fe^2+^ in a broader pH.^340^, ^341^ The nanoplatforms containing Cu and Fe ions showed higher apoptosis owing to the massive ROS production and accelerated Fe^2+^ regeneration in presence of Cu^+^.^342^ For alleviating the side effects of radiosensitizers on healthy tissues, Zhang et al.^343^ synthesized Cu_2_(OH)PO_4_ (Cu_I_) which is activated in massive hydrogen peroxide concentration of tumors. Normoxia of the healthy tissues deactivates the chemodynamic performance of the nanoparticles by transforming Cu_I_ to the neutral Cu_II_. In contrast, Cu_II_ conversion to Cu_I_ is facilitated within hypoxic condition of the tumor.^344^ Therefore, the difference of lethal ROS production is high under tumor microenvironment versus healthy tissues under radiation therapy. Pure copper‐based NPs and superparamagnetic magnetite has low atomic number, therefore, engineering them with high‐Z elements brings a boosted dose enhancement effect.^345^, ^346^ MOFs of Fe and Cu elicited radiosensitizing activity and GSH depletion and ferroptosis‐inducer.

Hauser et al.^347^ functionalized the surface of Fe_3_O_4_ NPs with TAT for a combination therapy with Fenton reaction and RT. TAT is a cell penetrating protein which also allows endosomal escape. Fe_3_O_4_/TAT/RT, RT, and Fe_3_O_4_/TAT showed 48%, 72.6% and 90.4% survival fraction at 72 h on A549 cells, respectively. SPIONS have been employed in other studies using RT, but their role in inducing additional cytotoxic mechanisms has not been fully studied.^348^, ^349^ Also, a further increase in the probability of cell death was achieved by using siRNA delivery to down‐regulate HIF‐1α expression, because knocking out HIF‐1α can stimulate the degradation of PARP‐1.^350^ Increasing tumor oxygenation using photothermal therapy^351^ has also been combined with Fenton reaction‐like approaches.

As fenton‐reaction assisted radiation therapy is affected by the amount of GSH, methods for manipulating it's biosynthesis instead of GSH removal. Biosynthesis of GSH is initiated by ɣ‐glutamylcysteine production from glutamate and cysteine and next transforming it to GSH. Enzymes named glutamate‐cysteine ligase (GCL) and GSH synthetase (GSS), respectively, are responsible for accelerating the mentioned reactions. Commercialized compounds such as L‐buthionine sulfoximine (BSO) are able to remove GCL and GSH thereof, leading to sensitize the cells against radiation therapy.^352^, ^353^

### 5.9 Trimodal therapy

The success of the synergistic combinations of bimodal therapy, led to the emergence of a triple or trimodal therapy combination. The design of nanostructures incorporating three separate modes of therapy is always challenging, however it may also lead to higher efficacy. Due to the toxicity concerns faced by RT used at a much higher dosages, trimodal therapy may ensures that a low dose is sufficient to obtain an increased in cell death. Therefore, in this section we discuss some examples of trimodal approaches including radiosensitizing nanoparticles.

#### 5.9.1 Chemotherapy/PDT/RT

As previously discussed, drugs were initially used to sensitize the cancer cells to ionizing radiation. However, the complex physicochemical properties of tumors can restrict drug penetration into the tumor, and consequently decrease their radiosensitizing effect.^354^ In NP‐based drug delivery, the enhanced permeability and retention (EPR) effect takes advantage of the hyperpermeable vasculature and poor lymphatic drainage as a passive delivery method. The leaky characteristics of the vasculature are aggravated upon PDT using low light doses, which increased the accumulation of liposomal doxorubicin in the tumor.^355^ Damage to the vasculature after PDT is responsible for the improved delivery of nano‐agents, allowing deeper penetration and more homogenous distribution.^356^ The optimized dose of laser energy should be used to avoid any unwanted damage to surrounding tissue. Fan et al.^357^ synthesized up‐conversion Gd‐mesoporous silica (radiosensitizer) loaded with hematoporphyrin (PS) and docetaxel (cytotoxic drug). The tumor was entirely eliminated by day 2, which indicated the successful triple combination of chemo/PDT/RT.

#### 5.9.2 Chemotherapy/PTT/RT

The synergistic effects of PTT/RT and chemotherapy/RT were discussed in earlier sections. However, the advantage of performing trimodal therapy is that there may be additional synergy between PTT and chemotherapy. PTT can increase the penetration of drug‐containing nanocarriers into the tumor by disrupting the ECM proteins and increasing vascular permeation.^358^ Moreover, PTT facilitates NP uptake by the tumor cells^359^ and enhances the toxicity of the drug by modifying cellular pathways.^360^ This combination has shown some promise in clinical applications, with some pathologically confirmed complete remissions having been documented.^361^ Namely, Li et al.^362^ utilized mesoporous silica‐coated GNPs plus berberine as a chemotherapy drug. The nanosystem was able to prevent tumor volume progression for more than 20 days in vivo. In addition, berberine protected the mice from RT side effects as shown by quantification of intestinal fatty‐acid binding protein (iFABP) and D‐amino‐oxidase (DAO).

Stimuli responsive drug release has enabled a precise drug delivery to the tumor. Active drug release occurs under biochemical, physical and chemical stimuli. Acidic microenvironment of the tumor is able to stimulate drug release in pH sensitive systems. Wang et al.^363^ fabricated Janus NPs composed of triangular GNPs/mesoporous silica and modified with PEG‐FA. Tirapazamine, a hypoxia‐sensitive prodrug, was loaded and released in the acidic pH of the tumor. There was twice as much γ‐H2AX (an indicator of DSBs) in the triple therapy than every single and double therapy. In addition, temperature increase for PTT could be leveraged for an on‐demand drug release, simultaneously. Namely, Kuang et al.^364^ synthesized Gd_2_Hf_2_O_7_ NPs and decorated them with polydopamine, PEG, and RGD. This system could be loaded with cisplatin, and smart drug release was triggered by pH and heat stimuli. The survival ratio was considerably diminished in the trimodal therapy compared with either bimodal therapy, or the therapies used alone. Mesoporous‐structured NPs have been used for two decades as drug delivery vehicles.^365^ Ma et al.^366^ fabricated mesoporous bismuth NPs as a photothermal agent and a radiosensitizer with drug loading capacity. A good photothermal conversion efficiency (η = 48.5%) coupled with pH and NIR dual responsive drug release boosted radiosensitizer‐mediated cell death. Song et al.^367^ produced a hollow porous palladium nanostructure for carrying X‐ray absorbing iodine derivatives, plus DOX, as a triple chemo‐photo‐ radiotherapy agent. Variable drug release profile as a function of pH plus 36.9% photothermal conversion efficiency produced only 14% cell survival. DOX monotherapy is accompanied severe fatigue, muscle weakness and body wight loss.^368^ Combination of DOX and RT will decrease the systemic adverse effects of it. Sun et al.^369^ fused erythrocyte and cancer cell membranes onto coated‐gold nanocages for PTT/RT and DOX drug delivery. The body weight of the animals decreased considerably with soluble DOX injection, and the tumor growth inhibition was not successful. In contrast, the combined therapy showed a body weight increase as well as a massive reduction of tumor volume. Homologous targetability of cancer cells relies on the ability of cancer cells to aggregate by the means of surface molecules such as epithelial cell adhesion molecule, N‐Cadherin, galectin‐3.^370^ The time‐consuming coating procedure and sampling from the patient in clinics will make this targeting method hard to translate in clinics.

#### 5.9.3 PTT/PDT/RT

Due to the inability of RT to destroy massive tumors, the combination of multiple therapies may be preferable. Meanwhile, PTT has shown promise as an adjunct therapy in clinical studies due to the above‐mentioned synergistic pathways.^258^ Adding PDT to therapies that utilize photonic sources to produce ROS along with radiosensitization is logical.

To this end, Qiu et al.^371^ produced PVP functionalized with ultrathin W_18_O_49_ nanowires, for triple PTT/RT/PDT therapy. In the multitherapy group, the tumor volume was reduced to zero at 5 days post‐irradiation with X‐rays (6 Gy, 5 min) and laser (980 nm, 1.2 W/cm^2^). Luo et al.^372^ used silica‐coated Au nanorods conjugated to hematoporphyrin photosensitizer and europium bromoacetate (EuBA) scintillator. In this system, low dose laser (808 nm, 0.8 w/cm^2^) and X‐ray (2 Gy) irradiation killed the cells and reduced the tumor volume. In a study by Xu et al.,^373^ hyaluronic acid‐modified Au nanocages, obtained by a redox reaction on Ag nanocubes, were used as a PTT/PDT/RT triple agent. Due to the presence of HA as a targeting ligand, which allowed high accumulation of NPs in the tumor site, a good reduction in tumor size was observed.

In conclusion, combination therapy involves numerous different techniques that can be used together for cancer treatment, while also minimizing conventional therapeutic resistance. However, accurate understanding of the mechanisms underlying combination therapies is essential for accelerating their clinical translation. When combination therapy is compared with the constitutive methods used alone, the best combination of modalities may be selected.^374^ Recently emerging laboratory methods, such as organoid culture models may be used to recapitulate tumor‐associated components and uncover hidden effects.^375^

## 6 CONCLUSIONS AND PERSPECTIVES

The emergence of high‐Z NPs as radiosensitizers with multifunctional properties is expected to revolutionize conventional radiation therapy. Elucidation of the exact mechanisms behind each therapeutic modality, while understanding their synergistic effects is crucial for their clinical translation. Insufficient knowledge about the role of photothermal therapy as an immunological mediator has restricted our deeper insight into radiation and photothermal combination therapy mechanisms.

Despite some successes, research on the combination of radiosensitizers and newly introduced therapeutic modalities, such as sonodynamic therapy and gene delivery has remained scarce. The delivery of oxygen and novel methods for in situ oxygen production through catalytic reactions, have been recently explored to boost RT efficacy. However, the use of PFC‐based compounds, regardless of their drawbacks, could accelerate the clinical translation.

The discovery of the best combination of modalities and their many variable parameters (e.g., duration, dosage, and sequence of administration) is challenging to design trials for eventual clinical application. The use of tumor mimicking laboratory technologies such as spheroids, microfluidics, and artificial intelligence could all be beneficial to tackle the aforementioned problems.

Theranostic agents can combine both multiple therapeutic effects as well as imaging modalities into a single system. These can provide on‐demand tracking of the NP biodistribution. Nano‐radiosensitizers often show high X‐ray attenuation which could be utilized as CT‐scan imaging contrast agent. High gap amongst k‐edge of high‐Z nanoparticle and biological tissue enables photon counting CT imaging. Engineering nano‐radiosensitizers could add additional MRI imaging modality via surface modification (e.g., with Motexafin Gadolinium), doping (e.g., with Mn and Gd) and heterostructures (e.g., with SPIONS). Also, quantum dots based on high‐Z materials with fluorescence imaging capability could be used as theranostic agent.

Immunotherapy and radiotherapy have been used clinically, however, they suffer from low efficiency on several metastatic cancers or dense tumors. Amongst studied combination, adjuvant effect of nano‐radiosensitizers in immunotherapy has more potential to be used in clinics. Polymeric nanoparticles modified with maleimide functional group were able to collect DAMPs. Smart high‐Z nano‐radiosensitizers with DAMPs capturing activity and active targeting of dendritic cells may increase the combination therapy efficacy. Also, immune checkpoint inhibitor therapy mediates T‐cells toward tumors. High‐Z element containing MOF has shown promising in the delivery of immunotherapeutic antibodies targeting immune checkpoints.

Chemical enhancement utilizes catalytic root for overcoming the energy barrier of ROS production. Nano‐radiosensitizer combination with PDT and chemodynamic therapy in a single system is categorized under chemical enhancement. Beyond ROS, catalytic root could be leveraged in order to produce free radicals based on alkyl, chlorine, semiquinone radicals. Moreover, further studies on catalytic depletion of GSH and SOD for impairing the antioxidative system of the malignant cells. Fenton/Fenton‐like catalytic reactions deplete GSH through valence transition reaction. Passive activity of Fenton reactions cause toxicity on healthy tissues, therefore, tumor activated Fenton reaction is preferred.

The reciprocal effect of nano‐radiosensitizer‐cell interaction and combination method has been poorly understood. Shedding light on these effects allows to uptake more nanoparticle in combination versus monotherapy (as shown for PTT). In addition, Subcellular localization of the nano‐radiosensitizers is amongst major factors governing the dose enhancement ratio. Therefore, directing the nano‐radiosensitizers from blood stream to the subcellular level (e.g., nucleus, mitochondria) brings the final treatment at a lower dose of therapy.

## AUTHOR CONTRIBUTIONS

**Mohammad Varzandeh:** Conceptualization (equal); data curation (equal); formal analysis (equal); investigation (equal); methodology (equal); resources (equal); software (equal); visualization (equal); writing – original draft (equal). **Leila Sabouri:** Data curation (equal); resources (equal); writing – original draft (equal). **Vahid Mansouri:** Data curation (equal); visualization (equal); writing – original draft (equal). **Maliheh Gharibshahian:** Methodology (equal); resources (equal); writing – original draft (equal). **Nima Beheshtizadeh:** Conceptualization (equal); investigation (equal); methodology (equal); project administration (equal); supervision (equal); visualization (equal); writing – original draft (equal); writing – review and editing (equal). **Michael R. Hamblin:** Funding acquisition (equal); validation (equal); writing – review and editing (equal).

## FUNDING INFORMATION

This study was not funded by any funding institute. Michael R. Hamblin was supported by US NIH grants R01AI050875 and R21AI121700.

## CONFLICT OF INTEREST

Michael R. Hamblin declares the following potential conflicts of interest. Scientific Advisory Boards: Transdermal Cap Inc, Cleveland, OH; Hologenix Inc. Santa Monica, CA; Vielight, Toronto, Canada; JOOVV Inc., Minneapolis‐St. Paul MN; Consulting; USHIO Corp, Japan; Sanofi‐Aventis Deutschland GmbH, Frankfurt am Main, Germany. The other authors declare that they have no competing interests.

### PEER REVIEW

The peer review history for this article is available at https://publons.com/publon/10.1002/btm2.10498.

## Data Availability

The data that support the findings of this study are available from the corresponding author upon reasonable request.
